# A novel BH3 mimetic Bcl-2 inhibitor promotes autophagic cell death and reduces in vivo Glioblastoma tumor growth

**DOI:** 10.1038/s41420-022-01225-9

**Published:** 2022-10-29

**Authors:** Seyma Calis, Berna Dogan, Serdar Durdagi, Asuman Celebi, Ozlem Yapicier, Turker Kilic, Eda Tahir Turanli, Timucin Avsar

**Affiliations:** 1 Neuroscience Laboratory, School of Medicine, Bahcesehir University, Istanbul, Turkey; 2 Department of Molecular Biology, Genetics and Biotechnology Graduate Program, Istanbul Technical University, Istanbul, Turkey; 3 Department of Medicinal Biochemistry, Bahcesehir University School of Medicine, Istanbul, Turkey; 4 Computational Biology and Molecular Simulations Laboratory, Department of Biophysics, School of Medicine, Bahcesehir University, Istanbul, Turkey; 5 School of Pharmacy, Bahcesehir University, Istanbul, Turkey; 6 Department of Pathology, Bahcesehir University School of Medicine, Istanbul, Turkey; 7 Department of Neurosurgery, Bahcesehir University School of Medicine, Istanbul, Turkey; 8 Department of Molecular Biology and Genetics, Faculty of Engineering and Natural Sciences, Acıbadem University, Istanbul, Turkey; 9 Department of Medical Biology, Bahcesehir University School of Medicine, Istanbul, Turkey

**Keywords:** Drug development, Cancer models

## Abstract

Anti-apoptotic members of the Bcl-2 family proteins play central roles in the regulation of cell death in glioblastoma (GBM), the most malignant type of brain tumor. Despite the advances in GBM treatment, there is still an urgent need for new therapeutic approaches. Here, we report a novel 4-thiazolidinone derivative BH3 mimetic, BAU-243 that binds to Bcl-2 with a high affinity. BAU-243 effectively reduced overall GBM cell proliferation including a subpopulation of cancer-initiating cells in contrast to the selective Bcl-2 inhibitor ABT-199. While ABT-199 successfully induces apoptosis in high *BCL2*-expressing neuroblastoma SHSY-5Y cells, BAU-243 triggered autophagic cell death rather than apoptosis in GBM A172 cells, indicated by the upregulation of *BECN1, ATG5*, and *MAP1LC3B* expression. Lc3b-II, a potent autophagy marker, was significantly upregulated following BAU-243 treatment. Moreover, BAU-243 significantly reduced tumor growth in vivo in orthotopic brain tumor models when compared to the vehicle group, and ABT-199 treated animals. To elucidate the molecular mechanisms of action of BAU-243, we performed computational modeling simulations that were consistent with in vitro results. Our results indicate that BAU-243 activates autophagic cell death by disrupting the Beclin 1:Bcl-2 complex and may serve as a potential small molecule for treating GBM.

## Introduction

Many cellular processes contribute to carcinogenesis, including dysregulated apoptotic cell death. A key mechanism related to apoptosis is the B-cell lymphoma-2 (Bcl-2)-mediated intrinsic apoptosis pathway [1]. Bcl-2 family proteins are well-known regulators of mitochondrial apoptosis, also known as the intrinsic apoptosis pathway. Bcl-2 family proteins have become targets for cancer therapy, along with other central regulators [2], because of their roles in apoptosis and autophagy, and their association with resistance to chemotherapy [3].

Several Bcl-2 inhibitors have been extensively studied against various cancers. N-acylsulfonamide derivative ABT-737 was one of the pioneering Bcl-2 inhibitors [4]. However, because of the poor bioavailability and low solubility of ABT-737, it was structurally modified and ABT-263 (Navitoclax) was developed. Clinical phase I–II studies of ABT-263 revealed that following Bcl-xl inhibition, patients had low platelet counts, and thrombocytopenia, as a rather severe side effect [1]. Further structural modifications were performed to ABT-263 and ABT-199 (Venetoclax) was developed [5] to overcome thrombocytopenia. ABT-199 showed 100-fold increased selectivity for Bcl-2 over Bcl-xl or Bcl-w and it was found to be more effective against Chronic Lymphocytic Leukemia (CLL) with milder cases of thrombocytopenia [6].

There have been several studies conducted with various Bcl-2 inhibitors and their effects on glial tumors. Glioblastoma multiforme (GBM) is the most aggressive type of brain tumor. It has been shown that ABT-737 promotes apoptosis in GBMs when used alone or in combination with other cytotoxic drugs [7]. It was also demonstrated that a small organic compound called HA14-1, which binds to Bcl-2 and especially inhibits its interaction with Bax, reduces the proliferation of GBM cells in vitro and in vivo [8]. Separately, a combinational treatment strategy using ABT-263 and PI3K inhibitor GDC-0941, showed that resistance to apoptosis was overcome via downregulation of *MCL1* [9]. Another promising Bcl-2 inhibitor gossypol showed anti-tumor effects on temozolomide-resistant GBM tumorspheres [10]. Anti-proliferative effects of temozolomide and gossypol combination were also investigated in another study and found that combined treatment inhibits GBM tumor angiogenesis, invasion, and proliferation [11]. Although these small molecules are found to be effective against gliomas, because of the low expression of *BCL2*, low molecular affinity towards Bcl-2, gained resistance, and severe side effects such as nausea, diarrhea, and low white blood cell counts [12], there is still a need for a safe small molecule with a high affinity to Bcl-2 to effectively induce cell death in GBM.

In a previous study [13], our group identified seven potential Bcl-2 inhibitor small molecules computationally and showed their Bcl-2 inhibition potential, as well as their effect on cell proliferation. Three of those were found to have better solubility and a high-affinity binding potential to the Bcl-2. In this study, we further analyzed these three molecules named BAU-58 (AJ-292/12931005), BAU-243 (AN-698/40780701), and BAU-199 (AG-205/12549135). We found that BAU-243 was the most effective even at lower concentrations when compared to BAU-58 and BAU-199.

Here, we further elucidated the mechanism of action of a novel Bcl-2 inhibitor BAU-243 on GBM cells, in vitro and in vivo. In addition, we examined the molecular mechanisms of BAU-243 using computational molecular simulation techniques and obtained results that were consistent with the experimental results. We show that rather than inducing intrinsic apoptotic cell death through inhibition of Bcl-2, BAU-243 disrupts the interaction between Beclin 1 and Bcl-2 by binding to the BH3 domain binding groove of Bcl-2 with a high affinity, and induces autophagic death of GBM cells.

## Results

### BAU-243 significantly reduced the proliferation of GBM cells

**Fig. 1:**
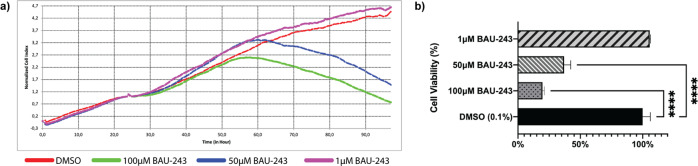
BAU-243 inhibited GBM cell proliferation. **a** Normalized cell index of A172 cells treated with DMSO (0.1%), 100 µM, 50 µM, and 1 µM BAU-243. Electrical impedance measurements were taken every 15 min for 96 h. Treatment was done at 24 h after cell seeding. **b** Percentage cell viability of A172 cells treated with BAU-243. DMSO treated group was accepted as 100%, and cell viability of each condition after treatment at 24, 48, and 72 h were normalized to the DMSO control group. Data are expressed as mean ± SD. Statistical analysis was performed using a two-tailed Student’s *t*-test (*n* = 3). *p < 0.05, **p < 0.01, ****p < 0.0001.

To identify novel compounds that can bind to Bcl-2 protein with higher affinity than previously clinically tested BH3 mimetics, such as ABT-199, we previously conducted a computational screening study [13]. Among the compounds that are found to efficiently bind to Bcl-2 computationally, the ones with high in vitro inhibition potential of Bcl-2, efficient anti-proliferative effect on GBM cells, and sufficient solubility to ensure better bioavailability were chosen to be studied further. Of tested compounds, BAU-243 demonstrated the most consistent anti-proliferative effect at 50 µM and 100 µM but not at 1 µM (Fig. 1a), and it significantly reduced the viability of A172 GBM cells (Fig. 1b). Therefore, we decided to further elucidate the anti-proliferative mechanism of action of BAU-243 on GBM cells.

### Survival of GBM cells and tumor-initiating stem cells were reduced by BAU-243

**Fig. 2:**
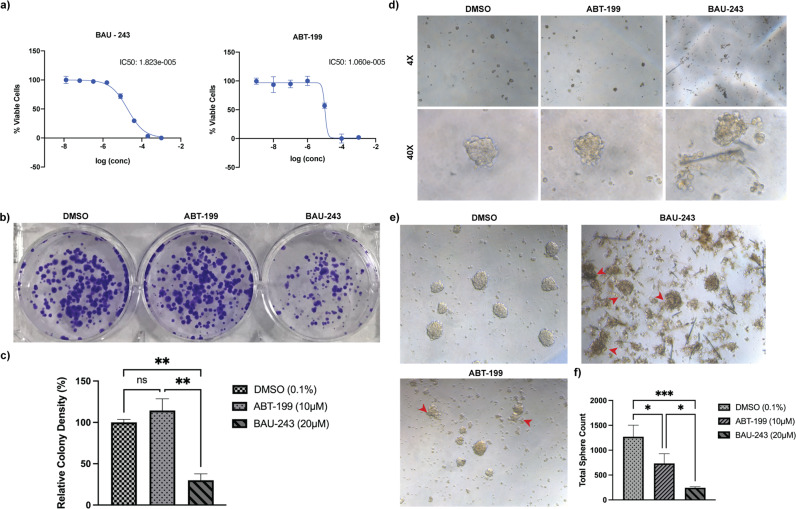
Tumor-initiating cancer cells were reduced by BAU-243. **a** IC_50_ calculation of BAU-243 and ABT-199 treated A172 cells. Concentrations for BAU-243 are ranging between 1 mM to 2.56 nM, diluted 5-fold for each concentration. Concentrations for ABT-199 are ranging between 1 mM to 1 nM, diluted 10-fold for each concentration. Percentage viable cell measurements were normalized to DMSO (0.1%) control group. IC_50_ values were calculated by Prism 9 software. **b** Colony formation assay of YKG1 cells. Images of DMSO (0.1%), ABT-199(10 µM), and 243 (20 µM) treated YKG1 colonies on day 8 (*n* = 2). Cells were fixed with ice-cold methanol and stained with 0.05% Crystal Violet. **c** Percentage relative colony density normalized to the DMSO control group. Colony density was determined as pixel counting by Adobe Photoshop. **d** Initial tumorsphere formation of YKG1 cells on the 4th day after treatment with DMSO (0.1%), ABT-199 (10 µM), or 243 (20 µM). Images are shown as 4X and 40X magnifications. **e** Tumorsphere formation of YKG1 cells on day 8 after treatment. Images are shown as 20X magnification. Red arrows indicate disintegrated spheres. **f** Total sphere count of DMSO (0.1%), ABT-199 (10 µM), or 243 (20 µM) treated cells on day 8. Data are expressed as mean ± SD. Statistical analysis was performed using an ordinary one-way ANOVA, together with Dunnett’s multiple comparisons test. *p < 0.05, **p < 0.01, ***p < 0.001, ns not significant.

To determine the IC_50_ values of BAU-243 and clinically tested BH3 mimetic ABT-199, we designed an experiment with A172 cells with concentrations ranging between 1 mM and 2.56 nM (Fig. 2a). IC_50_ value of the BAU-243 molecule was calculated as 18.2 µM and IC_50_ of ABT-199 molecule was found to be 10.6 µM. Hence, we decided to conduct the remaining in vitro experiments with concentrations of 20 µM and 10 µM for BAU-243 and ABT-199 respectively.

Testing the cytotoxicity of anticancer compounds with the colony formation assay provides crucial insights into the anti-proliferative potential of a candidate compound since only a portion of a cell population has the capacity for unlimited division [14]. To determine the clonogenic capacity of YKG1 GBM cells following treatment with either DMSO (0.1%), BAU-243, or ABT-199 for 72 h, we designed a colony formation assay. While ABT-199 did not exert any effect on YKG1 cells when compared to the DMSO control group, BAU-243 effectively reduced the proliferation of the cells (Fig. 2b). Statistical analysis showed that BAU-243 significantly diminished relative colony density (*p* < 0.01) (Fig. 2c), suggesting that BAU-243 has the capacity to inhibit the growth of sub-population cells that may serve as tumor-initiating cells.

It has been established that GBM stem cells are the source of resistance to chemotherapy and radiotherapy [15]. Therefore, targeting GBM stem cells as a form of GBM therapy has become an important approach in recent years. To determine the effect of ABT-199 and BAU-243 on GBM stem cells, we designed a tumorsphere formation assay with YKG1 cells. As early as the 4th day after treatment, while DMSO-treated cells formed spheres with clear and rigid edges, ABT-199 and BAU-243 treated spheres started to disintegrate (Fig. 2d). On the 8th day, spheres were observed more clearly, and dispersing of the spheres following BAU-243 treatment became more apparent, compared to ABT-199 treated spheres (Fig. 2e). Total sphere count revealed that anti-proliferative effect of BAU-243 on GBM stem cells was more significant than ABT-199 (Fig. 2f). Taken together, these results suggested that BAU-243 might be a more effective Bcl-2 inhibitor than ABT-199 for GBM treatment.

### BAU-243 promoted limited apoptosis in low Bcl-2-expressing GBM cells

**Fig. 3:**
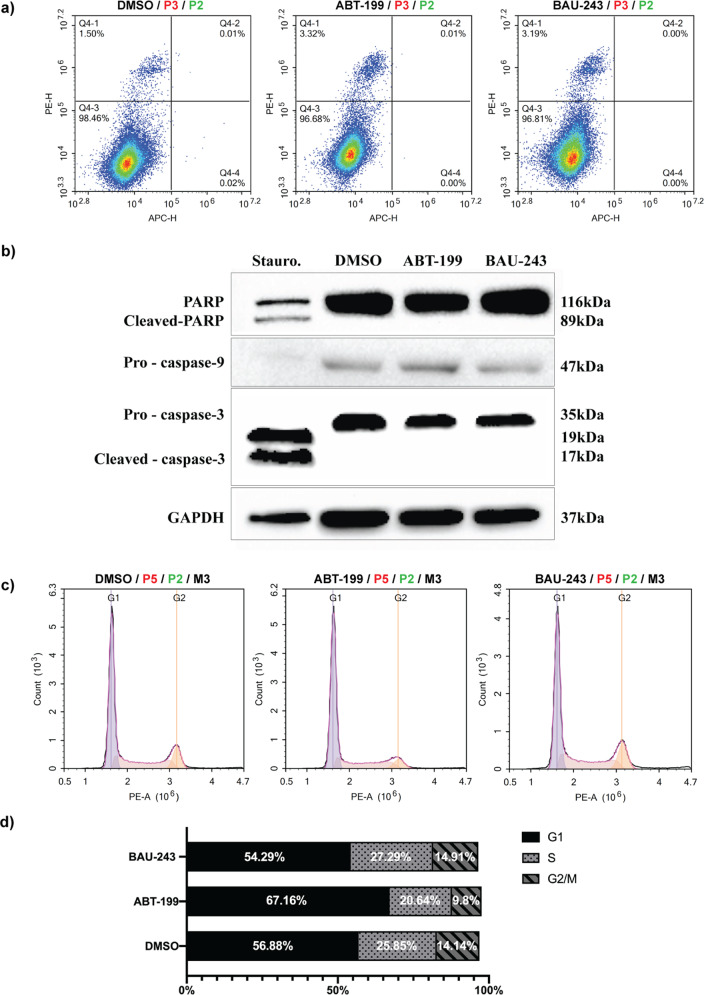
BAU-243 induced limited apoptosis and cell cycle arrest in low Bcl-2 expressing cells. **a** Flow cytometric apoptosis analysis of A172 cells by Annexin V/PI staining, treated with DMSO (0.1%), ABT-199 (10 µM), and BAU-243 (20 µM) for 72 h. Annexin V is detected on the APC channel, and PI is detected on the PE channel. The bottom left quadrant shows viable cells, the bottom right quadrant shows early apoptotic cells, the top right quadrant shows late apoptotic cells, and the top left quadrant shows necrotic/dead cells. **b** Western blots of apoptosis-related proteins PARP, cleaved-PARP, pro-caspase-9, and pro-caspase-3. Total protein was isolated from A172 cells. Cells were either treated with 1 µM of staurosporine for 4 h, DMSO, ABT-199, or BAU-243 for 72 h. 50 µg per lane was loaded. GAPDH was used as an internal control. **c** Cell cycle analysis of A172 cells with PI staining, treated with DMSO (0.1%), ABT-199 (10 µM), and BAU-243 (20 µM) for 72 h. PI is detected on the PE channel. **d** Cell cycle stage fractions of A172 cells, showing G1, S, and G2/M phases.

Bcl-2 inhibition is expected to trigger apoptosis in cancer cells because of its central role in the intrinsic apoptosis pathway [16]. To assess the number and fraction of apoptotic cells within the total cell population, we performed Annexin V / PI staining. Surprisingly, we did not observe any significant increase in the number of apoptotic A172 cells after 72 h of treatment with either DMSO (0.1%), ABT-199, or BAU-243 (Fig. 3a). We analyzed apoptosis-related proteins and observed that cleaved-PARP, a product of caspase-3 cleavage, was not expressed in A172 cells apart from the positive control group that was treated with Staurosporine, a known inducer of apoptosis (Fig. 3b). Moreover, pro-caspase-3 and pro-caspase-9 levels were downregulated only with the treatment of Staurosporine, indicating the cleavage of the full-length form to release the active form (Fig. 3b). To test any possible effects of ABT-199 and BAU-243 on cell cycle in A172 cells, we conducted a PI staining and determined the fraction of cells in each cell cycle stage. While ABT-199 caused an apparent G1 arrest of A172 cells following treatment, BAU-243 did not show any effect on the cell cycle (Fig. 3c, d).

### BAU-243 did not induce apoptosis in high Bcl-2 expressing SHSY-5Y cells despite the significant anti-proliferative effect

**Fig. 4:**
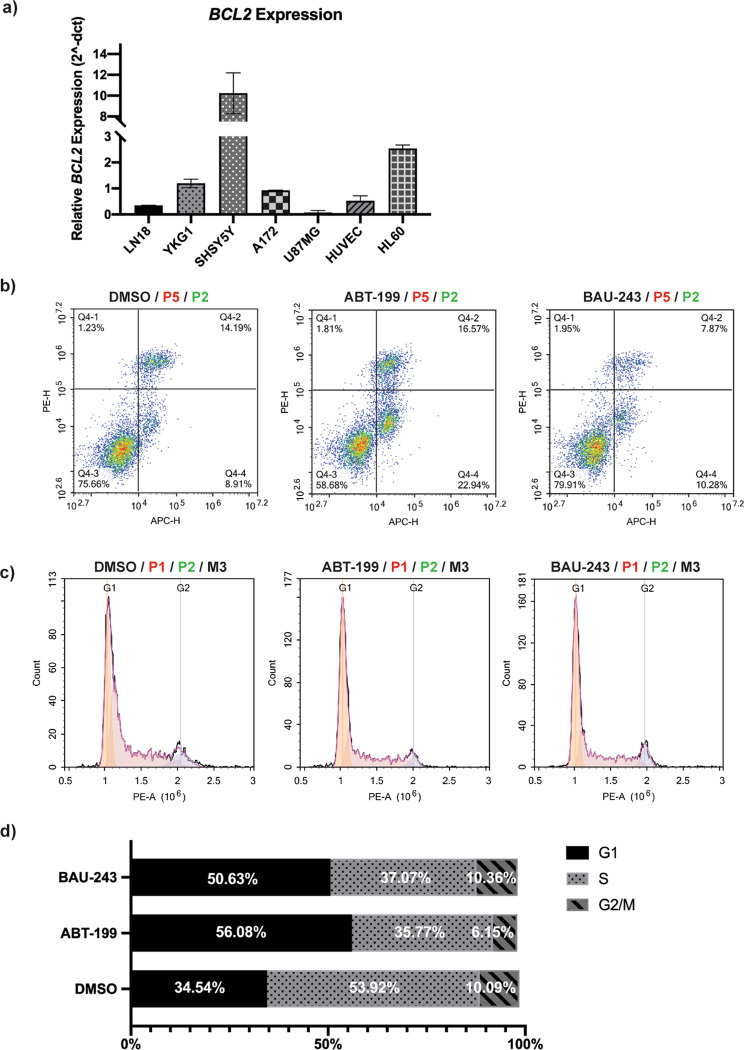
High Bcl-2 expressing cells were not significantly induced apoptosis upon BAU-243 treatment. **a** Quantitative RT-PCR of *BCL2* expression of different cell lines compared. cDNA was converted from 500 ng/µl of RNA. *GAPDH* was used as an internal control, and Cq values are normalized to the *GAPDH* expression of each cell line. 2^-dct^ was calculated (*n* = 2). **b** Flow cytometric apoptosis analysis of SHSY-5Y cells by Annexin V/PI staining, treated with DMSO (0.1%), ABT-199 (10 µM), and BAU-243 (20 µM) for 72 h. Annexin V is detected on the APC channel, and PI is detected on the PE channel. The bottom left quadrant shows viable cells, the bottom right quadrant shows early apoptotic cells, the top right quadrant shows late apoptotic cells, and the top left quadrant shows necrotic/dead cells. **c** Cell cycle analysis of SHSY-5Y cells with PI staining, treated with DMSO (0.1%), ABT-199 (10 µM), and BAU-243 (20 µM) for 72 h. PI is detected on the PE channel. **d** Cell cycle stage fractions of SHSY-5Y cells, showing G1, S, and G2/M phases.

Linked with the differences in *BCL2* expression among tissues, each cell type can respond to apoptotic insults differently. It has been shown that hematological cancers are more prone to BH3 mimetics-mediated mitochondrial apoptosis [17]. Therefore, we assessed the *BCL2* expression of several cell lines including GBM cell lines A172, YKG1, LN18, U87MG, neuroblastoma cell line SHSY-5Y, promyelocytic cell line HL-60, and endothelial cell line HUVEC (Fig. 4a). As predicted, while HL-60 had an abundant *BCL2* expression, *BCL2* expression of SHSY-5Y cells was 10-fold higher than A172 cells (Fig. 4a). We decided to replicate apoptosis and cell cycle analyses in the SHSY-5Y cell line. BAU-243 did not show any pro-apoptotic effects on these cells when compared to the DMSO control group, although ABT-199 significantly increased apoptosis (Fig. 4b). Unlike in A172 cells, both ABT-199 and BAU-243 induced G1 arrest in a similar fraction in the SHSY-5Y cell line, when compared to control group (Fig. 4c, d). Therefore, our results suggested that BAU-243 might be utilizing a different mechanism other than apoptosis to exert an anti-proliferative effect on GBM cells.

### BAU-243 promoted autophagic cell death through the disruption of Beclin 1 and Bcl-2 interaction, regardless of Bcl-2 expression level

**Fig. 5:**
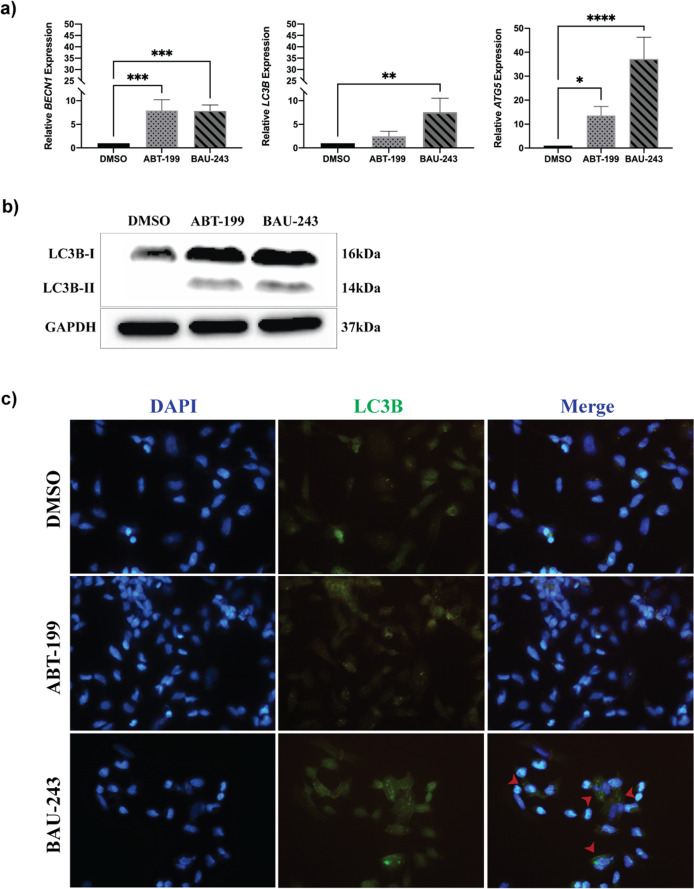
BAU-243 treatment induced autophagic cell death. **a** Quantitative RT-PCR of *BECN1, MAP1LC3B*, and *ATG5* of DMSO (0.1%), ABT-199 (10 µM), and BAU-243 (20 µM) treated A172 cells. Cq values are normalized to *GAPDH* expression, and 2^-ddct^ values were calculated (*n* = 4). **b** Western blotting of LC3B. 20 µg protein per lane was loaded. GAPDH was used as a control. **c** Immunocytochemistry fluorescence microscopy images (40X) of DMSO (0.1%), ABT-199 (10 µM), and 243 (20 µM) treated SHSY-5Y cells. DAPI is represented with blue and LC3B is represented with green. Red arrows indicate autophagosomes. Data are expressed as mean ± SD. Statistical analysis was performed with ordinary one-way ANOVA, together using Dunnett’s multiple comparisons test. *p < 0.05, **p < 0.01, ***p < 0.001, ****p < 0.0001.

Aside from its key roles in the mitochondrial apoptosis pathway, Bcl-2 also plays a part in the autophagic cell death pathway through its interaction with Beclin 1 [18]. Therefore, we sought to analyze expression levels of autophagy-related genes such as *BECN1, MAP1LC3B*, and *ATG5* in GBM cells. Quantitative RT-PCR results revealed that compared to the DMSO control group, ABT-199 and BAU-243 treatments significantly upregulated the expression of *BECN1* and *ATG5*. However, only BAU-243 significantly upregulated *MAP1L3CB* gene expression (Fig. 5a). Lc3b is the most extensively studied form within the Lc3 protein family, and the lipidated form of Lc3b-I, called Lc3b-II, is found on autophagosomes. It is therefore considered the active form of Lc3b-I, an indicator of autophagosome formation and induction of autophagy [19, 20]. We observed that BAU-243 treatment upregulated Lc3b-II protein expression, while DMSO and ABT-199 treatment did not exert any effect on Lc3b-II abundance (Fig. 5b). Additionally, we decided to investigate changes in Lc3b expression after 72 h of treatment with either DMSO (0.1%), ABT-199, or BAU-243. Immunocytochemistry analysis of Lc3b revealed that, while the control group had a small number of autophagic cells, mainly BAU-243 treated cells showed a clear staining pattern for Lc3b (Fig. 5c). Overall, these results suggest that BAU-243 triggers the activation of the autophagy pathway, rather than the apoptosis pathway, through inhibition of Bcl-2.

### BAU-243 mimics Beclin 1 BH3 domain and occupies the BH3 binding groove of Bcl-2

We previously identified the compound BAU-243 (named 243 in the previous study) by computationally screening the Specs SC small molecule library (https://www.specs.net/), which contains more than 200.000 compounds overall [13]. We then performed molecular docking and MD simulations to study its protein-ligand interactions. As a part of our earlier study, we utilized a crystal structure that contains an ABT-263 analog (PDB ID, 4LXD [5]). However, during the publication of our study, the crystal structure of Bcl-2 with ABT-199 was also demonstrated by Birkinshaw et al. [21]. In the current study, we performed molecular docking analyses for BAU-243, and utilized the crystal structure of Bcl-2 co-crystalized with ABT-199. Although the overall structure of Bcl-2 obtained with different inhibitors (i.e. structures with PDB IDs 4LXD and 6O0K) exhibited similar RMSD values, less than 1.00 Å, side chain rotations in the binding groove can affect the orientation of the ligand. We employed a different and more computationally expansive docking algorithm, QPLD, in which the partial charges of ligands could be calculated more accurately employing quantum mechanics (QM) (i.e., 6-31 G*/LACVP* basis set, B3LYP density functional). As a result, we obtained a different binding pose for BAU-243 compared to our previous study. We performed a re-docking analysis for co-crystalized ABT-199 by the QPLD approach and revealed that the obtained pose was similar to the crystal docking pose (RMSD between two poses is 1.12 Å). Our experimental data suggested that BAU-243 disrupted the interaction between Beclin 1:Bcl-2. Therefore, we carried out MD simulations for Bcl-2 in complex with Beclin 1 BH3 domain to determine critical Bcl-2 residues that might be interacting with the BH3 domain of Beclin 1. We sought to detect whether BAU-243 can also interact with these critical residues of Bcl-2 and could prevent the binding of Beclin 1 BH3 domain to the Bcl-2 hydrophobic groove. MD simulations were also performed for ABT-199 using the docking pose obtained by QPLD as an initial structure to determine whether this selective inhibitor and the suggested compound interact with the same critical residues of Bcl-2.

**Fig. 6:**
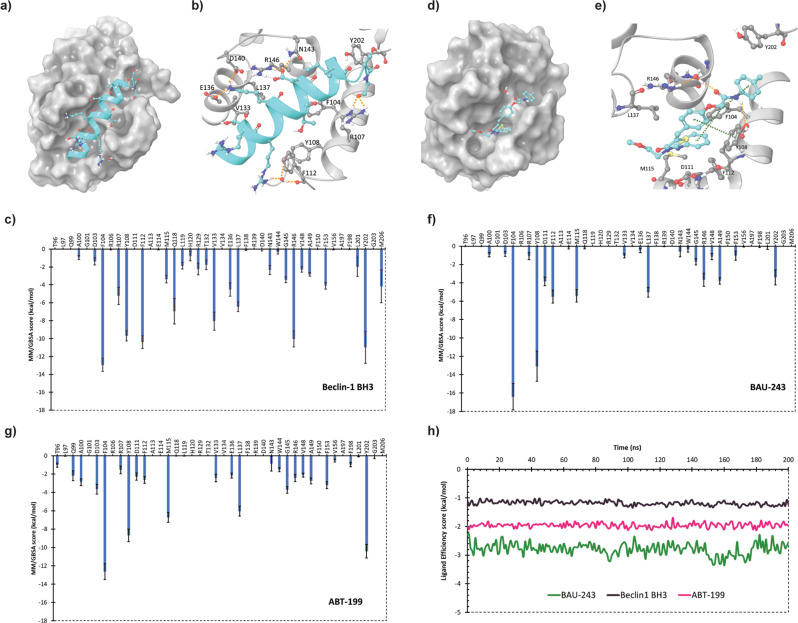
BAU-243 interacted with BH3 binding domain of Bcl-2 protein. **a** Bcl-2 in complex with Beclin 1 BH3 domain with Bcl-2 represented as gray colored surface. Water molecules and residues of Beclin 1 BH3 are displayed with the ball and stick representation with C atoms colored in dark cyan, O atoms in red, N atoms in dark blue, and polar H atoms in white. Orange dashed lines indicate hydrogen bonds. **b** 3D representation of interactions between Beclin 1 BH3 and Bcl-2 with Bcl-2 residue C atoms represented in gray and other atoms colored as in a. **c** Bcl-2 residues contribution plot for the estimated free energy of Beclin 1 BH3 and Bcl-2 complex calculated by MM/GBSA approach. **d** Bcl-2 in complex with BAU-243 with Bcl-2 represented as gray colored. Atoms in the ligand molecule are colored as explained in a. **e** 3D representation of interactions between BAU-243 and Bcl-2 with Bcl-2 residue C atoms represented in gray and other atoms colored as in (**a**). Orange dashed lines indicate hydrogen bonds while green dashed lines indicate π-π stacking interactions. **f** Bcl-2 residues contribution plot for the estimated free energy of BAU-243 and Bcl-2 complex calculated by MM/GBSA approach. **g** Bcl-2 residues contribution plot for the estimated free energy of ABT-199 and Bcl-2 complex calculated by MM/GBSA approach. **h** Ligand efficiency scores for Beclin 1 BH3, BAU-243, and ABT-199 were estimated throughout MD time.

Our molecular modeling simulations indicate that Y108, F112, R107, E136, D140, N143, and R146 residues of Bcl-2 form hydrogen bonding interactions or water-bridged contacts with BH3 of Beclin 1 (Fig. 6a, b). Based on the simulation results, E136 and R146 were involved in multiple hydrogen bonding interactions (Supplementary Fig. S1a). The interactions R146 formed were maintained throughout the whole MD simulation time while E136 formed less well-maintained hydrogen bonds (78% and 35% of MD time, Supplementary Fig. S1a). Hydrogen bonds D140 and N143 formed with BH3 of Beclin 1 were also persistent for almost all MD time (94% and 98% of MD time, respectively as seen in Supplementary Fig. S1a). On the other hand, the water bridge interactions were not very persistent (maintained less than 50% of MD time). F104, F112, and Y202 were involved in the formation of hydrophobic interactions with the BH3 domain of Beclin 1 which were well-preserved (Supplementary Fig. S1b, c). We have also computationally determined the contribution of these residues as well as the other residues in the binding pocket to estimate the free energy of binding for complex formation by an MM/GBSA approach (Fig. 6c). We observed that hydrophobic F104 was the residue with the highest contribution followed by Y202, F112, R146, and Y108. BAU-243 forms hydrogen bonds with Y108 that were maintained for most of the MD simulation time (73% of MD time, Supplementary Fig. S2a), and two water bridges with R146 (persistent for 46% and 29% of MD time, Supplementary Fig. S2a), two of the critical residues for Beclin 1 BH3 binding to Bcl-2. Via the phenyl moieties of BAU-243, it was forming persistent hydrophobic contacts with F104 and Y108 (Supplementary Fig. S2b, c). F104 was involved in π-π stacking interactions with two phenyl rings of BAU-243 (persistent for 74% and 60% of MD time, Supplementary Fig. S2a). Though the π-π stacking interaction with Y108 was not very persistent, this residue was still involved in the formation of hydrophobic contacts with BAU-243. When the contribution of Bcl-2 residues to the estimated free energy of binding of BAU-243 was computed, F104 was found to be the residue with the highest contribution. Y108, F112, R146, and Y202 also contributed substantially to estimated free energy as in the case of Beclin 1 BH3 binding. M115, L137, and D11 were other residues contributing to the BAU-243 binding. Conversely, the reference compound ABT-199 forms hydrogen bond and/or water bridge interactions with D103, D107, N143, and Y202, while also forming π-π stacking interactions with F104, Y108, and Y202 (Supplementary Fig. S3a). It also occupies the BH3 domain binding groove of Bcl-2 (Supplementary Fig. S3b). The Bcl-2 residues that contribute significantly to the estimated free energy of binding for ABT-199 were F104, Y202, Y108, M115, and L137 (Fig. 6g). A detailed protein-ligand analysis for this reference compound based on our MD simulations was also performed (Supplementary Fig. S3). Lastly, we compared the computed binding free energy values by MM/GBSA for Beclin 1 BH3, BAU-243, and ABT-199. We divided the calculated values by the number of nonhydrogen atoms to calculate the interaction energy per atom (i.e., ligand efficiency scores) as the BH3 domain of Beclin 1, BAU-243 and ABT-199 have a different number of atoms (average values provided in Table S3). The ligand efficiency score of the suggested compound was more negative, indicating a better efficiency score for BAU-243 compared to BH3 of Beclin 1, and an even better efficiency score than ABT-199 (Fig. 6h). Therefore, our modeling studies suggest that BAU-243 could occupy the Bcl-2 binding groove and it form stable interactions with critical residues of Bcl-2 that are involved in Beclin 1 BH3 binding.

### Low dose BAU-243 reduced tumor growth in vivo compared to other BH3 mimetics

**Fig. 7:**
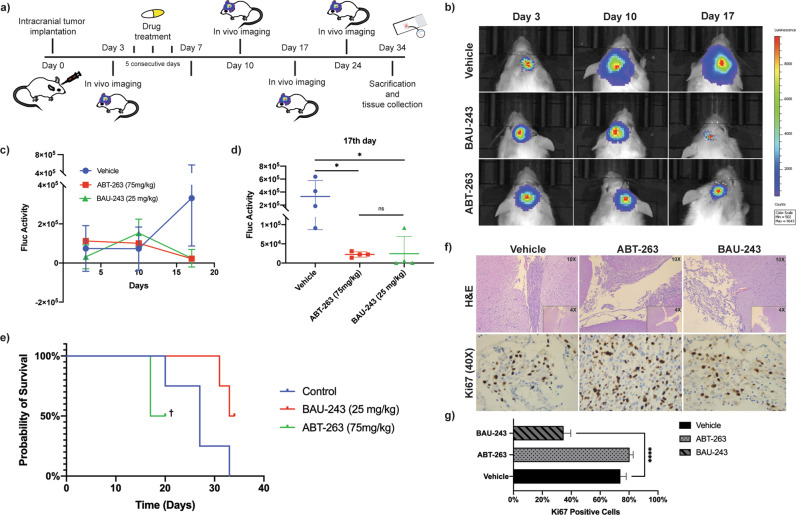
BAU-243 significantly inhibited in vivo tumor growth. **a** Summary of experimental design. 1 × 10^5^ Firefly Luciferase expressing U87MG GBM cells inoculated into the brains of NOD/SCID Gamma mice with a stereotactic frame. From day 3 through day 7, each treatment group (*n* = 4) received 5 doses of either vehicle, ABT-263 (75 mg/kg), or BAU-243 (25 mg/kg). Tumor growth was visualized and followed by in-vivo imaging, and on day 34 experiment was ended to obtain brain tissues. **b** In vivo imaging of Vehicle, ABT-263 (75 mg/kg), and BAU-243 (25 mg/kg) treated mice. Representative one mouse from each group is shown. **c** Tumor growth Fluc activity of Vehicle, ABT-263 (75 mg/kg), and BAU-243 (25 mg/kg) treated mice. **d** Fluc activity of each animal in each group on the 17th day. **e** Kaplan Meier curve of the probability of survival for each group. † Remaining two animals from ABT-263 treated group had to be euthanized due to ethical concerns. **f** H&E and Ki67 staining of brain sections from each treatment group. H&E images were shown as both 4X and 10X magnifications. Ki67 images were shown at 40X magnification. **g** Percentage of Ki67 positive cells. The percentile of Ki67 positive cells relative to all stained cells is calculated from three independent images for each group. Data are expressed as mean ± SD. Statistical analysis was performed using ordinary one-way ANOVA, together with Dunnett’s multiple comparisons test. *p < 0.05, ****p < 0.0001.

To reveal in vivo potential of BAU-243, we orthotopically inoculated U87MG-FmC to NOD/SCID Gamma mice (Fig. 7a). Control group received only the vehicle, while treatment group received ABT-263, and the test group received BAU-243 (*n* = 4 for each group). Bioluminescence Firefly Luciferase activity revealed that tumor volume of BAU-243 treated mice significantly decreased when compared to control and ABT-263 treated groups (Fig. 7b). Mean Fluc activity in different groups indicated that, although a relatively lower dose of BAU-243 was administered compared to ABT-263, it significantly shrank overall tumor volume (Fig. 7c, d), as well as prolonged overall survival in test group animals (Fig. 7e). However, Fluc activity on day 24 post-treatment showed that tumor volumes of each group increased again (Supplementary Fig. S4), suggesting that animals could benefit more from continuous treatment, and resistant cancer cells might be eradicated with repeated treatments. For further analysis, the brains of animals from each group were obtained, paraffin-embedded, and sectioned for immunohistochemical analysis with Hematoxylin and Eosin and Ki67 staining (Fig. 7f). Statistical analysis of Ki67 proliferation index (*n* = 3) revealed that BAU-243 significantly decreased tumor progression (*p* < 0.0001) when compared to control and ABT-263 treated groups (Fig. 7g).

## Discussion

In this study, we investigated novel Bcl-2 inhibitory small molecules and identified the BAU-243 compound as an effective candidate for exerting an anti-proliferative impact on GBM cells. Having a similar IC_50_ value with a clinically available Bcl-2 inhibitor ABT-199, BAU-243 demonstrated a remarkable anti-proliferative effect on the cancer sub-population that is considered a true representative of tumor-initiating GBM stem-like cells. Due to its well-known role in the intrinsic apoptosis pathway, we expected to observe an increased apoptotic response among BAU-243 treated cells following Bcl-2 inhibition. However, possibly because of the low *BCL2* expression of GBM cells, we did not detect significantly increased apoptosis. Similarly, there was no apparent effect on the cell cycle as well following BAU-243 treatment. Although we did observe apoptosis in high *BCL2-*expressing neuroblastoma cells, similar to GBM cells, BAU-243 did not induce apoptosis of neuroblastoma cells as well. We further confirmed the lack of apoptosis by western blots, showing that both ABT-199 and BAU-243 failed to induce apoptosis of low *BCL2-*expressing GBM cells. Because the Beclin 1:Bcl-2 complex has a role in autophagy, we hypothesized that BAU-243 might be disrupting the interaction between the two and triggering autophagy. As predicted, following BAU-243 treatment autophagy-related genes were upregulated, and an abundant Lc3b-II expression was observed by immunocytochemistry and western blot. We also showed a disruption of Beclin 1:Bcl-2 interaction through molecular modeling studies. Finally, the BAU-243 treatment demonstrated significant tumor growth reduction in vivo.

The potential of Bcl-2 inhibitors to treat various cancers has been established in recent years. Gossypol (AT-101) tested in GBM treatment, binds to BH3 domains of Bcl-2, Bcl-xl, and Mcl-1, and is reported to efficiently inhibit in vitro and in vivo growth of glial tumors [11]. A phase I trial included patients that are newly diagnosed with GBM, who received radiotherapy and temozolomide, to determine the maximum tolerated dose of gossypol [22]. Later, a Phase II trial was designed and the effects of gossypol on patients with progressive or recurrent GBM was studied [23]. According to the results of a Phase II study with 56 patients, monotherapy of gossypol resulted in 1.87 months of progression-free survival, and 5.9 months of overall survival of GBM patients [24], which is not considered to be significant given the fact that recurrent GBM patients’ overall survival is already measured between 5.4–9.9 months [25]. Moreover, due to the high affinity of gossypol to Bcl-xl, similarly to ABT-737 and ABT-263, it can be considered that the low platelet count might be a severe side effect and therefore limits its concentration for administration. Taking these results into account, there is still a need for a Bcl-2 inhibitor that can selectively inhibit Bcl-2, and result in successful clinical outcomes.

In accordance with our previous study, which demonstrated the high affinity and inhibitory capacity of BAU-58, BAU-199, and BAU-243 compounds to Bcl-2 [13], we further analyzed their in vitro antiproliferative potential. Solubility of a compound is essential to conduct reliable in vitro assays, and to demonstrate in vivo efficiency and bioavailability [26]. In addition to their better solubility, among the three compounds, BAU-243 exerted a significant anti-proliferative effect on GBM cells. Therefore, we further elucidated the mechanism of action of BAU-243, a 4-thiazolidinone derivative. There have been several studies determining the anticancer effects of 4-thiazolidinone derivatives. More commonly studied derivatives are thiazolidine and 4-thiazolidinone, and it has been shown that these molecules have anticancer effects on several cancers including chronic myeloid leukemia, lung carcinoma, breast cancer, prostate cancer, oral cancer, colorectal cancer, neuroblastoma, and glioma [27–32]. While thiazolidine and 4-thiazolidinone derivatives exhibit different mechanisms of action to exert their anticancer effects, only a few of many studies reported their relation with autophagy pathways [33, 34]. Therefore, it would yield crucial insights to elucidate the relation between thiazolidine derivatives and the autophagy mechanism.

One of the greatest challenges of in vitro drug screening is to efficiently mimic tumor microenvironment. There have been many assays developed to serve different purposes, such as clonogenic and spheroid-based assays. The clonogenic assay is established as a 2D culture, and it determines the sub-populations that are capable of self-sustaining which makes it an efficient way to assess cytotoxicity [35]. BAU-243 demonstrated a significant effect of reducing colony numbers in a colony formation assay to assess tumor cell proliferation, indicating its potential capacity for reducing cancer progression. Another way to test cytotoxicity is spheroid-based assays, which are 3D cultures, and they can recapitulate a population of cells termed cancer stem cells. A few of the many advantages of spheroid assays are the representation of interactions between cells with each other and the microenvironment, demonstration of proliferation gradients, and tumor mass-like necrotic cores [36]. In our tumorsphere assays, BAU-243 exerted a significant anti-proliferative effect on GBM cells, supporting its anti-tumor potential.

Due to its central role in mitochondrial apoptosis, Bcl-2 inhibition is considered to induce a malfunctioning apoptosis pathway, which is one of the most common ways for cancer cells to escape death [37]. However, BAU-243 did not induce apoptotic cell death in GBM. Furthermore, there has been no apparent dysregulation of the cell cycle upon BAU-243 treatment to induce cancer cell death. It has been established in the literature that; low *BCL2* expression might decrease the yield of BH3 mimetic-driven induction of the intrinsic apoptosis pathway [38–40]. Therefore, when we assess *BCL2* expression levels among several cell lines, we observed that GBM cells have low *BCL2* expression, while neuroblastoma cells express *BCL2* abundantly. In light of these data, upon treatment of neuroblastoma cells with BAU-243, we expected to induce apoptosis. However, BAU-243 did not trigger intrinsic apoptosis in high *BCL2-*expressing neuroblastoma cells as well. Although, BAU-243 showed a similar effect with ABT-199 on the cell cycle, by promoting a G1 arrest.

To explain the mechanism of action of BAU-243 and its anti-proliferative effect on GBM cells, we hypothesized that another cell death mechanism related to Bcl-2, other than mitochondrial apoptosis that is normally associated with Bcl-2 inhibition, might be in action. Bcl-2 has been shown to inhibit autophagy through its interaction with Beclin 1, a key player in autophagy initiation [41]. Some other BH3 mimetics are shown to disrupt the interaction between Beclin 1:Bcl-2 and induce autophagy of various cancer cells, namely gossypol, obatoclax, and ABT-737 [4, 42]. Furthermore, some studies reported that Beclin 1 independent autophagy induction through Bcl-2 inhibitors, is observed in diseases like breast cancer and colorectal cancer by ABT-199 and obatoclax, respectively [43, 44]. BAU-243 treatment upregulated gene expression levels of *BECN1*, as well as other autophagy-related genes such as *ATG5*, and *MAP1LC3B*. Atg5 plays an important role in initial autophagosome formation, moreover, it acts as an inter-player between autophagy and apoptosis [18, 45, 46]. Both upregulated gene expression levels and abundant Lc3b expression in cells shown by western blots and immunocytochemistry upon BAU-243 treatment indicate that autophagy is induced through BAU-243 treatment.

Molecular modeling studies also indicated that BAU-243 interacts with critical residues of Bcl-2 that plays role in the binding of Beclin 1 BH3 domain, showing that it can hinder Beclin 1:Bcl-2 complex formation, freeing Beclin 1 to activate the autophagy pathway. When we checked the crucial residues of Beclin 1 (BH3 domain): Bcl-2 interaction residues it is found that F104, Y108, F112, R146, and Y202 contribute significantly to the binding interactions. Interestingly, all of the residues (i.e., F104, Y108, F112, R146, and Y202) that contribute significantly to Beclin 1:Bcl-2 binging were also found to be critical in the binding of BAU-243 to Bcl-2.

In conclusion, our data indicate a therapeutic potential for a novel Bcl-2 inhibitor BAU-243, thiazolidinone derivatives, for GBM treatment through induction of autophagic cell death. Our data also suggests that the mechanism of action of 4-thiazolidinone derivatives might be through autophagy, which can be elucidated in the near future. Later studies could also benefit from systems-level in vivo characterization of toxicity, tolerance, and combinatorial drug dynamics to evaluate further translational potential of BAU-243 in the treatment of gliomas.

## Materials and methods

### Cell culture

Cell lines U87MG-FmC, A172, LN18 (kind gifts from Dr. Tugba Bagci-Onder), YKG1 (#TKG0453, Tohoku University, Japan), SHSY-5Y (#CRL-2266, ATCC, Manassas, VA, USA), HUVEC (#PCS-100-013, ATCC, Manassas, VA, USA), and HL60 (#CCL-240, ATCC, Manassas, VA, USA) were fed with Dulbecco’s Modified Eagle Medium (DMEM) High Glucose (4.5 g/l) with L-glutamine and sodium pyruvate (Capricorn Scientific GmbH, Ebsdorfergrund, Germany), supplemented with 10% heat-inactivated Fetal Bovine Serum (FBS) (Capricorn Scientific GmbH, Ebsdorfergrund, Germany) and 1% Antibiotic/Antimycotic solution (Capricorn Scientific GmbH, Ebsdorfergrund, Germany). Cell lines were sub-cultured twice a week. Cells were treated with the compounds for 72 h unless otherwise stated. For cell culture studies all experiments were studied in triplicates and replicated three times.

### Preparation of compound solutions

Small molecule 243 (BAU-243, AN-698/40780701), for in vitro studies was purchased from SPECS (Zoetermeer, The Netherlands). ABT-199 (Venetoclax - #HY-15531) and ABT-263 (Navitoclax - #HY-10087) were purchased from MedChemExpress (New Jersey, NY, USA). Compounds were dissolved in 100% DMSO to generate either 100 mM or 50 mM solutions to use as the main stock. Vortexing, mild sonication, and/or heating of the compound facilitated the dissolving of the molecules. To obtain working concentrations, serial dilutions with high-glucose DMEM supplemented with L-glutamine, sodium pyruvate, and FBS were performed.

### xCELLigence proliferation assay and IC_50_ calculation

xCELLigence RTCA DP system and E-plate 16 PET (#300600890, Agilent, Santa Clara, CA, USA) were used for cell proliferation assays. Before starting cytotoxicity assays, a cell seeding density experiment was performed. Each condition was studied as triplicates. After deciding the appropriate seeding number, cytotoxicity assays were performed with the appropriate seeding density of cells following the prior seeding number calculation. For the cytotoxicity assay, following 24 h of initial cell seeding, the assay was paused, and treatments were applied. The experiment was resumed for another 72 h. For IC_50_ determination of BAU-243, concentrations ranging from 2.56 nM to 4 mM were used, and for ABT-199 concentrations ranging from 1 mM to 1 nM were used. IC_50_ values after 72 h of treatment of each compound were calculated with Prism 9 software by linear regression analysis (GraphPad Software, San Diego, CA, USA).

### Colony formation assay

The assay was designed in 6-well plates. Cells were seeded at a density of 500 cells/well and 2 ml of the medium was added to each well. The next day, cells were treated in duplicates with either DMSO (0.1%), ABT-199 (10 µM), or Bcl-2 inhibitor BAU-243 (20 µM). On days 10-12, cells were washed twice with 1X PBS and fixed with ice-cold methanol for 5 min. 1% aqueous crystal violet solution (#V5265-250mL, Sigma–Aldrich, St. Louis, MO, USA) was diluted with 1X PBS to 0.25% and added to the cells. Cells were incubated for 30 min at room temperature. Crystal violet was removed, cells were washed under tap water, and were left to dry. Wells were scanned, and images were analyzed using Adobe Photoshop CC (Version 14.0) (San Jose, CA, USA).

### Tumorsphere assay

Cell pellets were dissolved in Neurobasal Medium (Gibco #21103049, Thermo Fisher Scientific, Waltham, MA, USA) supplemented with 1X L-glutamine (#XC-T1755, BioSera, France), 1X B27 supplement (Gibco #17504044, Thermo Fisher Scientific, Waltham, MA, USA), and 1X N2 supplement (Gibco #17502048, Thermo Fisher Scientific, Waltham, MA, USA). Cells were then seeded onto 25 ml ultra-low attachment cell culture flasks (#4616, Corning, NY, USA) at 100,000 cells/ml in 5 ml of medium. 20 ng/ml bFGF (Gibco #PHG0024, Thermo Fisher Scientific, Waltham, MA, USA) and 20 ng/ml EGF (Gibco #PHG0314, Thermo Fisher Scientific, Waltham, MA, USA) were added to the cells. Either DMSO (0.1%), ABT -199 (10 µM), or Bcl-2 inhibitor BAU-243 (20 µM) were added to the media on day 0. Media and growth factors were renewed every three days. Cells were propagated for 8–10 days. Cell clusters containing more than 50 cells were counted as a sphere. Counting and validations of tumorspheres were performed according to the formula recommended by Stem Cell Technologies [47].

### Annexin V apoptosis assay

Apoptosis was analyzed by BioLegend APC Annexin V / PI staining kit (#640932, San Diego, CA, USA). Instructions from the manufacturer were followed. Cells were incubated in dark with 5 µl PI and 2.5 µl Annexin V for 15 min at room temperature and analyzed using NovoCyte 3005 Flow Cytometry (Agilent, Santa Clara, CA, USA).

### Cell cycle analysis

For cell cycle analysis, Propidium Iodide (PI) (#A2261.0025, PanReac Quimica S.L.U, Spain) was used. Steps from a previously published method were used to assess changes in cell cycle progression [48]. Cells were analyzed with NovoCyte 3005 Flow Cytometer and Novo Express 1.5.0 Software (Agilent, Santa Clara, CA, USA).

### RNA Isolation and qRT-PCR

After treatment, RNA isolation was performed using a High Pure RNA Isolation Kit (#11828665001, Roche, Basel, Switzerland) and the kit’s instructions were followed. RNA concentrations were determined by Nabi UV/Vis Nano Spectrophotometer (MicroDigital Co., Seongnam, South Korea). RNA was converted to cDNA using an A.B.T. cDNA Synthesis Kit (#C03-01-20, Atlas Biyoteknoloji, Ankara, Turkey), and the kit’s instructions were followed. Bio-Rad T100 Thermal Cycler (#1861096, Bio-Rad, Hercules, CA, USA) was used to perform PCRs. Primers used for the study are listed in Supplementary Table S1. Reactions were run on a Bio-Rad CFX96 Touch Real-Time PCR Detection System (#1855196, Bio-Rad, Hercules, CA, USA). Samples were studied at least in duplicates, and Cq values were analyzed by delta-delta Ct analysis.

### Protein isolation and western blotting

After 72 h of treatment, cells were washed with ice-cold 1X PBS, and RIPA buffer was added to the cells. Cells were then collected with a scraper to isolate total protein. Protein concentrations were determined using Bradford assay (Protein Assay Dye Region Concentrate, #500-0006, Bio-Rad, Hercules, CA, USA). Proteins were separated by a 4–12% SDS PAGE gel (#1610175, Bio-Rad, Hercules, CA, USA) and transferred onto a nitrocellulose membrane (#1620150, Bio-Rad, Hercules, CA, USA). Primary and secondary antibodies and dilutions that are used in the study are listed in Supplementary Table S2. Visualizations were performed by Thermo Scientific Pierce ECL Western Blotting Substrate (#32106, Waltham, MA, USA) and imaging was done using a Bio-Rad ChemiDoc XRS + system, and ImageLab software (Bio-Rad, Waltham, MA, USA). ImageJ [49] was used for the analysis of the bands.

### Immunocytochemistry

The experiment was designed in 6-well plates with sterilized coverslips in each well. 50.000 cells/well were seeded and the next day cells were treated either with DMSO (0.1%), ABT-199 (10 µM), or Bcl-2 inhibitor BAU-243 (20 µM) for 72 h. Primary antibody against LC3B (#NB-1002220SS, Novus Biologicals, Littleton, CO, USA) and F(ab’)2-Goat anti-Mouse IgG (H + L) cross-adsorbed secondary antibody Alexa Fluor-488 (#A-11017, Invitrogen, Waltham, MA, USA) were used for the staining. Cell nuclei were stained with DAPI (10 mg/ml) (#A4099, AppliChem GmbH, Darmstadt, Germany). Cells were then observed with a Leica DM1000 fluorescent microscope (Leica Microsystems, Wetzlar, Germany).

### Molecular modeling studies

Two 3D structures of Bcl-2 target proteins were downloaded from the protein data bank (PDB): one co-crystallized with ABT-199 (PDB ID, 6O0K [21]), and another structure with Beclin 1 BH3 domain (PDB ID, 5VAU [50]). The structures were prepared using the protein preparation module [51] of Maestro modeling software. All ions and water molecules in the crystal structures were deleted. The missing side chain atoms were added and residues were numbered based on the crystal structure with PDB ID 6O0K. The unresolved loop region (between residues 35–91) was filled as described in our previous study [13]. The protonation states for side chains of residues were determined using the PROPKA module [52], while the ionization state for co-crystallized ligands was determined using Epik [53] at pH 7.4. To remove steric clashes and relax the structures, restricted geometry optimization and energy minimization were performed with OPLS3e [54] force field parameters.

The prepared 3D structures were used for molecular docking studies with co-crystallized ligands shaping the binding pocket boundaries. For docking studies, the quantum-polarized ligand docking (QPLD) algorithm [55, 56] of the Maestro molecular modeling package was utilized with partial charges of ligands computed at an accurate level using quantum mechanics (QM) calculations. The co-crystalized ligand ABT-199 was re-docked into the binding pocket to assess the docking procedure and obtain the QM charges of the ligand. BAU-243 was prepared with the LigPrep module [57] before docking to determine bond orders, atom types, and ionization states. The suggested BH3 mimetic compound was then docked into the binding sites of the prepared Bcl-2 structure, which was shaped based on the co-crystalized ABT-199. The top-scoring poses were selected for ABT-199 and BAU-243 at their respective binding sites. The docking pose obtained for ABT-199 was aligned with its co-crystalized binding mode and root mean square deviations (RMSD) between them were calculated to be 1.12 Å. Top-docking scored pose obtained at the binding site of the prepared Bcl-2 structure was chosen for BAU-243 and this complex structure was used in all-atom molecular dynamics (MD) simulations.

MD simulations were conducted for the complexes formed between Bcl-2 and BAU-243 as well as Bcl-2 and Beclin 1 BH3 domains. Desmond package [58] was utilized for MD simulations with OPLS3e [54] force field parameters. The partial charges of ligands calculated by Qsite [59, 60] during QPLD docking were used in MD simulations. The complex systems were positioned in cubic boxes of explicit TIP3P water with thicknesses of 10 Å, calculated from the protein surfaces. The systems were neutralized with the addition of Na^+^ and Cl^−^ ions and the concentration of solution systems was adjusted by 0.15 M NaCl solution. Before the production of MD simulations, minimization and equilibration steps were performed by using the default protocol of the Desmond package. The production simulations were run for 200 ns with trajectory files being collected at every 100 ps. Other details for production simulations were the same as the ones employed in our previous study [13].

Post-MD analysis was performed by using the collected trajectory files. The binding free energies for each molecule at their corresponding pockets were estimated by molecular mechanics/generalized Born surface area (MM/GBSA) approach utilizing the Prime module [61] of the Maestro molecular modeling package. 200 frames from each simulation were used for the calculations and average values, as well as standard deviations, were calculated from these frames. Additionally, residue-based MM/GBSA scores were calculated to determine the contribution of each binding pocket residue to estimated free energies. The collected trajectory files were also used to determine protein-ligand contacts observed during MD simulations and to determine the mobility of ligands at the binding sites of Bcl-2.

### Animal experiments

Animal experiments were conducted at an institutional animal facility following ethical codes, and ethical approval was obtained from Institutional Ethics Committee for Animal Experiments (2021/56) to perform all in vivo experiments. 6–8 weeks of age NOD SCID Gamma mice (*n* = 4/group) were used for the orthotopic mouse model. The exact sample size for animal studies was 48. All experiments were repeated 3 times. The sample size was determined by considering previous animal studies on xenografts and ethical concerns. Mice with motor disabilities, bleeding and edema formation after tumor injection were excluded from the study. No randomization of mice was used. Investigators were not blind to the mice groups. Intracranial injection procedure was performed as described before [62]. Briefly, Bregma was seen and a hole was burred with a drill at the top right of the bregma point (2 mm posterior and 1.5 mm lateral). U87MG GBM cells which are stably transfected with Luciferase (U87MG-FmC) were used for in vivo experiments, to be able to visualize tumor cells by bioluminescence. 7 µl of 100.000 U87MG-FmC cells in 7 µl PBS were injected intracranially through the hole with a 10 µl hamilton syringe (#80314, Hamilton Company, USA). The needle was inserted slowly into the 2 mm depth, and cells were injected a couple of inches per minute. After all, the cells were injected, the needle was withdrawn slowly and carefully to prevent leakage. Lastly, the incision was closed with a tissue stapler. On day 3, initial tumor growth was visualized by IVIS Spectrum In Vivo Imaging System (Perkin Elmer, Waltham, MA, USA). Follow-up in vivo images was taken on the 10th, 17th, and 24th days as well. Tested molecules were dissolved in 10% DMSO and 90% of corn oil. Control groups received 100 µl solvent, and test groups received 100 µl compound dissolved in 5% DMSO and 95% corn oil mixture by gavage, for five consecutive days between days 3 to 7. On the 34th day, the experiment was ended, perfusion was performed and the brains of the animals were paraffin-embedded for histological analyses.

### Histopathology studies

Paraffin-embedded tissues were sectioned at a 3 µm thickness. Hematoxylin / Eosin and Ki67 staining were performed as described before [63]. Ki-67 percentage values were calculated from three independent images for each condition.

### Statistical analyses

GraphPad Prism 9 was used for all statistical analyses. Data were expressed as mean ± SD. For multiple comparisons, ordinary one-way ANOVA with Dunnett’s multiple comparisons test was performed. The variance between groups being statistically compared was considered to be similar. For unpaired data, a two-tailed Student’s *t*-test was used. *p* < 0.05 was accepted as significant.

## Supplementary information

Authors consent to addition of Dr. Asuman Celebi as co-author to this manuscript

Original Data File

Supplementary Information

## Data Availability

The original data used to support the findings of this study will be provided by corresponding author upon reasonable request.
