# Supplementary material for: A novel BH3 mimetic Bcl-2 inhibitor promotes autophagic cell death and reduces in vivo Glioblastoma tumor growth
Source: Cell Death Discov. 2022 Oct 29;8:433. doi: 10.1038/s41420-022-01225-9 (PMC9617882; doi:10.1038/s41420-022-01225-9)

**Full uncut blots**

**Figure 3b PARP**


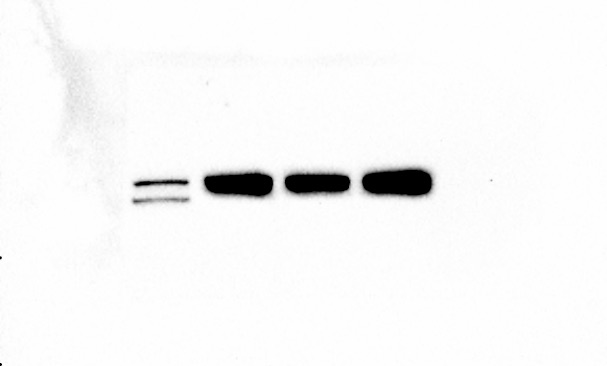


**Figure 3b Caspase-9**

Caspase-9 was blotted on the stripped membrane that was previously blotted with Caspase-3 (lower bands)


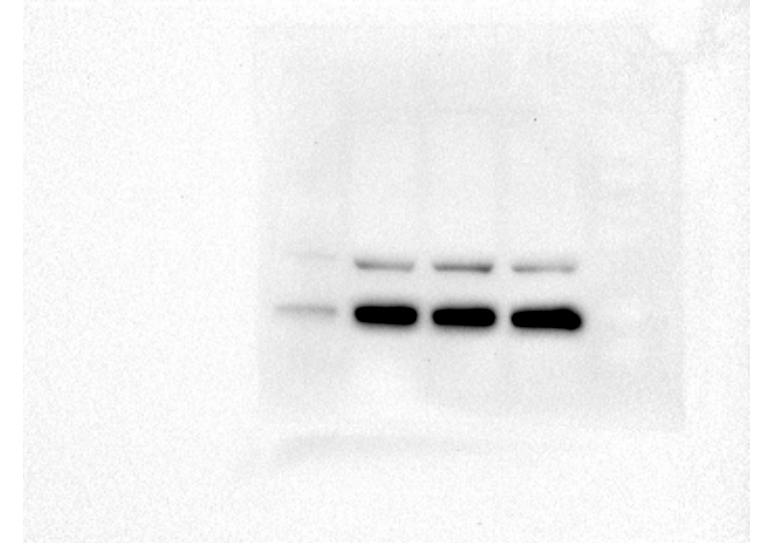


caspase-3

**Figure 3b Caspase-3**

**
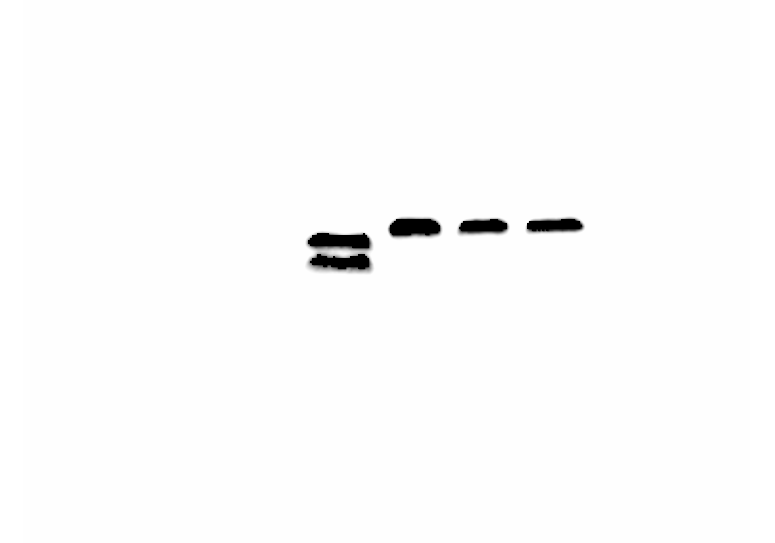
**

**Figure 3b GAPDH**


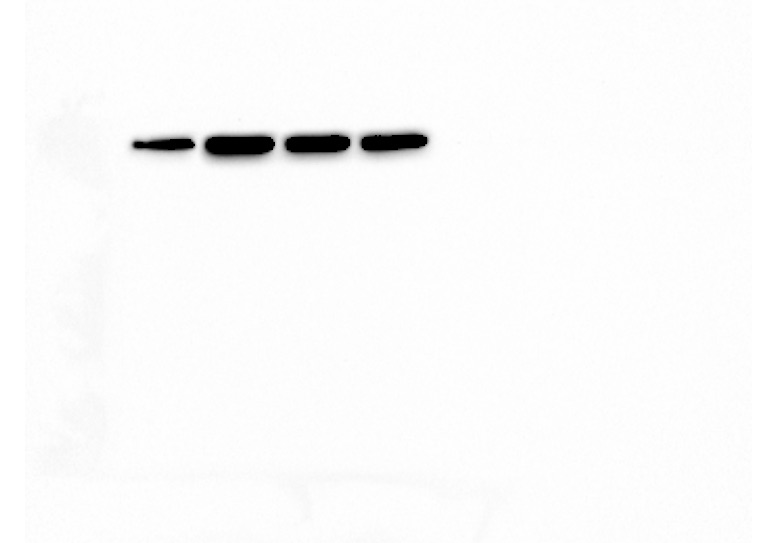


**Figure 5b LC3B**

**
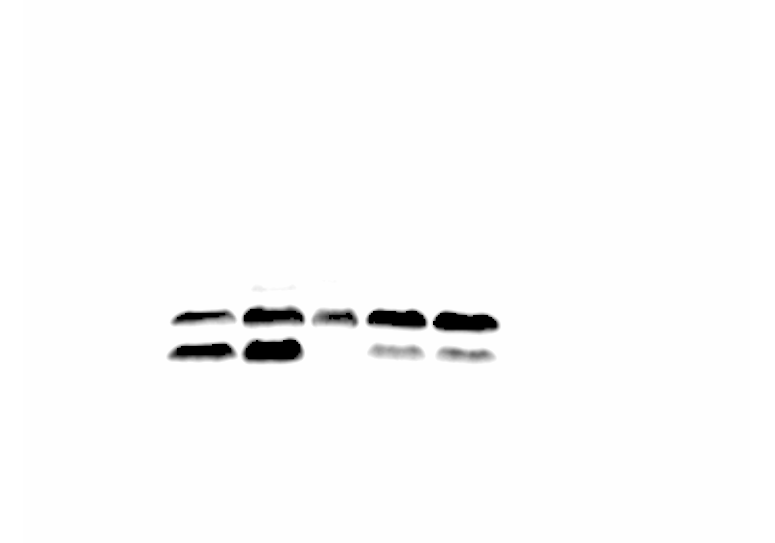
**

**Figure 5b GAPDH**


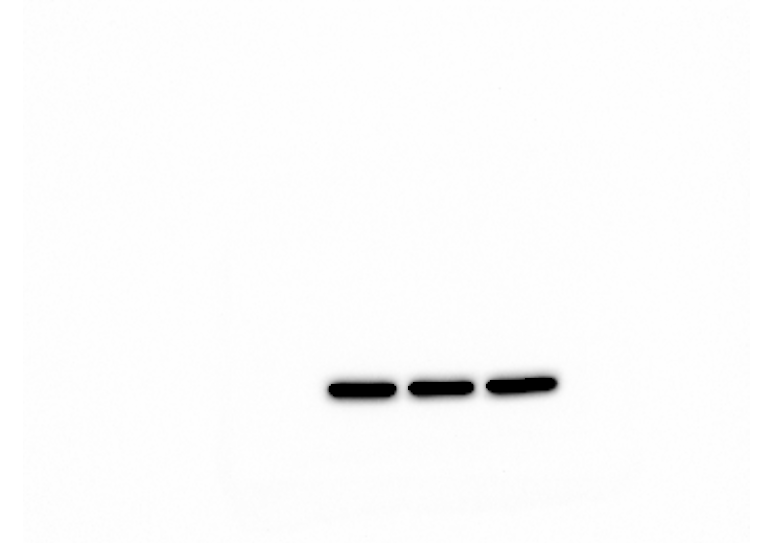

Supplement: Supplementary file 2 — Original Data File [file 41420_2022_1225_MOESM2_ESM.docx]
