# Supplementary material for: A novel BH3 mimetic Bcl-2 inhibitor promotes autophagic cell death and reduces in vivo Glioblastoma tumor growth
Source: Cell Death Discov. 2022 Oct 29;8:433. doi: 10.1038/s41420-022-01225-9 (PMC9617882; doi:10.1038/s41420-022-01225-9)
Supplement: Supplementary file 3 — Supplementary Information [file 41420_2022_1225_MOESM3_ESM.docx]

**Supplementary Information**

**A novel BH3 mimetic Bcl-2 inhibitor promotes autophagic cell death and reduces *in vivo* Glioblastoma tumor growth**

Seyma Calis^1,2^, Berna Dogan^3^, Serdar Durdagi^4,5^, Ozlem Yapicier^6^, Turker Kilic^7^, Eda Tahir Turanli^8^, Timucin Avsar^1,9,*^

^1^Neuroscience Laboratory, Health Sciences Institute, Bahcesehir University, Istanbul, Turkey; ^2^Department of Molecular Biology, Genetics and Biotechnology Graduate Program, Istanbul Technical University, Istanbul, Turkey; ^3^Department of Medicinal Biochemistry, Bahcesehir University School of Medicine, Istanbul, Turkey; ^4^Computational Biology and Molecular Simulations Laboratory, Department of Biophysics, School of Medicine, Bahcesehir University, Istanbul, Turkey; ^5^School of Pharmacy, Bahcesehir University, Istanbul, Turkey; ^6^Department of Pathology, Bahcesehir University School of Medicine, Istanbul, Turkey; ^7^Department of Neurosurgery, Bahcesehir University School of Medicine, Istanbul, Turkey; ^8^Department of Molecular Biology and Genetics, Faculty of Science and Letters, Acıbadem University, Istanbul, Turkey; ^9^Department of Medical Biology, Bahcesehir University School of Medicine, Istanbul, Turkey.


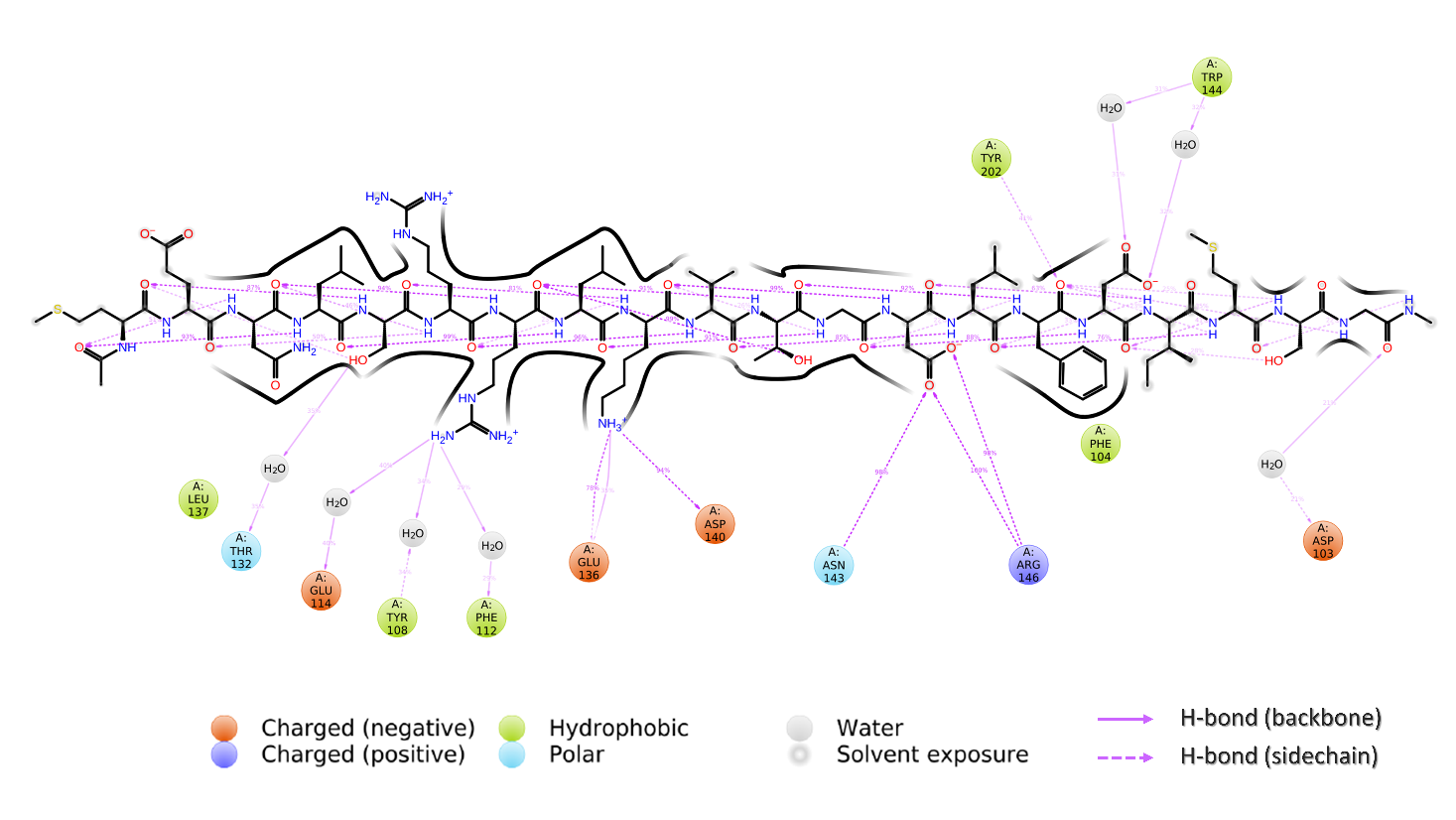


**A)**

**B) C)**


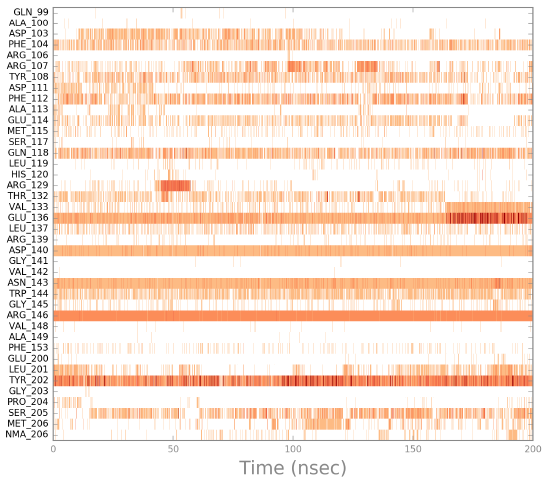

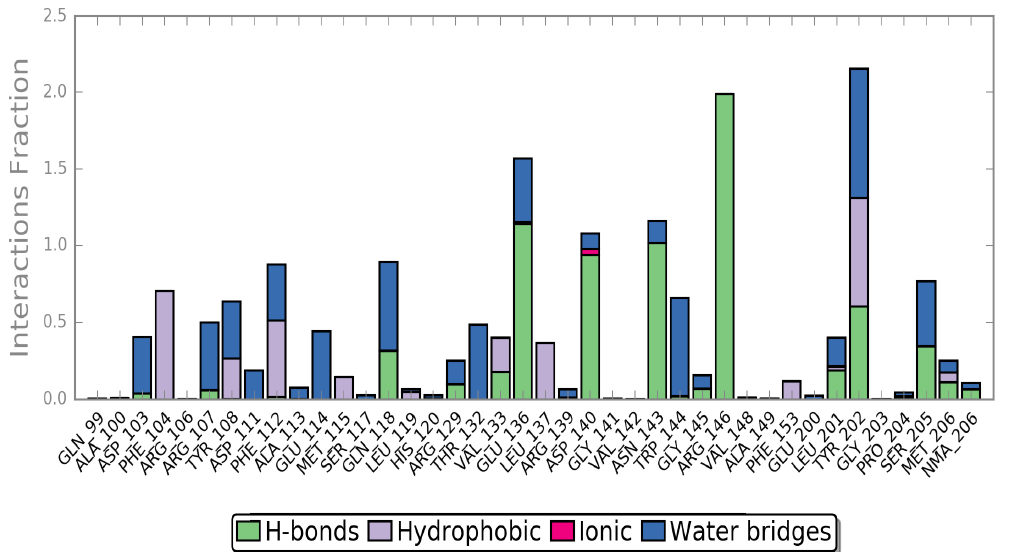

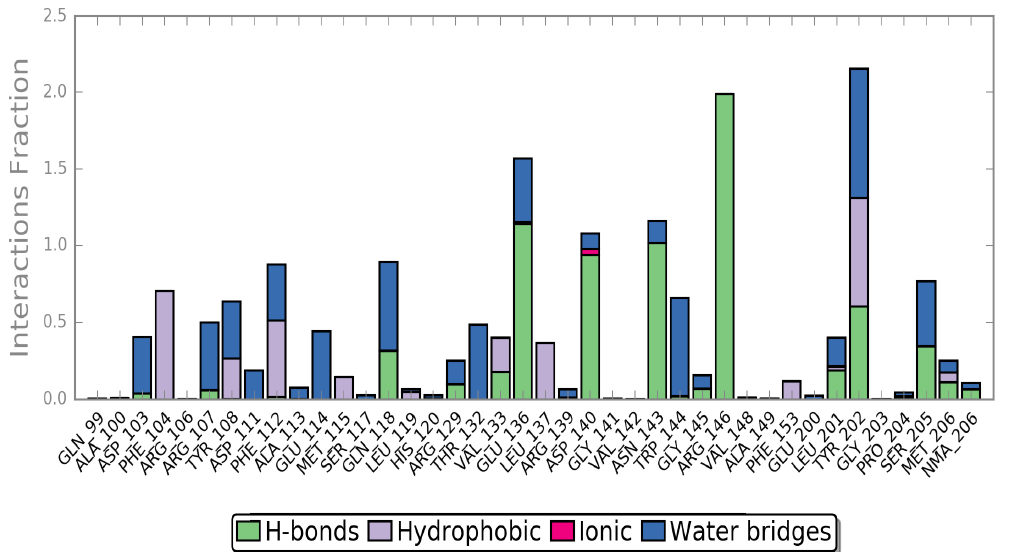


**Supplementary Figure S1** Protein-ligand interaction analysis for BH3 domain of Beclin 1 at Bcl-2 binding groove throughout MD simulation a) 2D interaction diagram with to what percentage of the simulation time the displayed interactions maintained are indicated. Cut-off value was taken as 20%. b) Histogram of non-covalent interactions, normalized over the course of trajectory. Values higher than 1.0 indicate protein residue make multiple contacts with the ligand. c) A timeline representation of the interactions for protein residues displayed in B. Darker colors denote the residue makes multiple contacts with the ligand.

**A)**


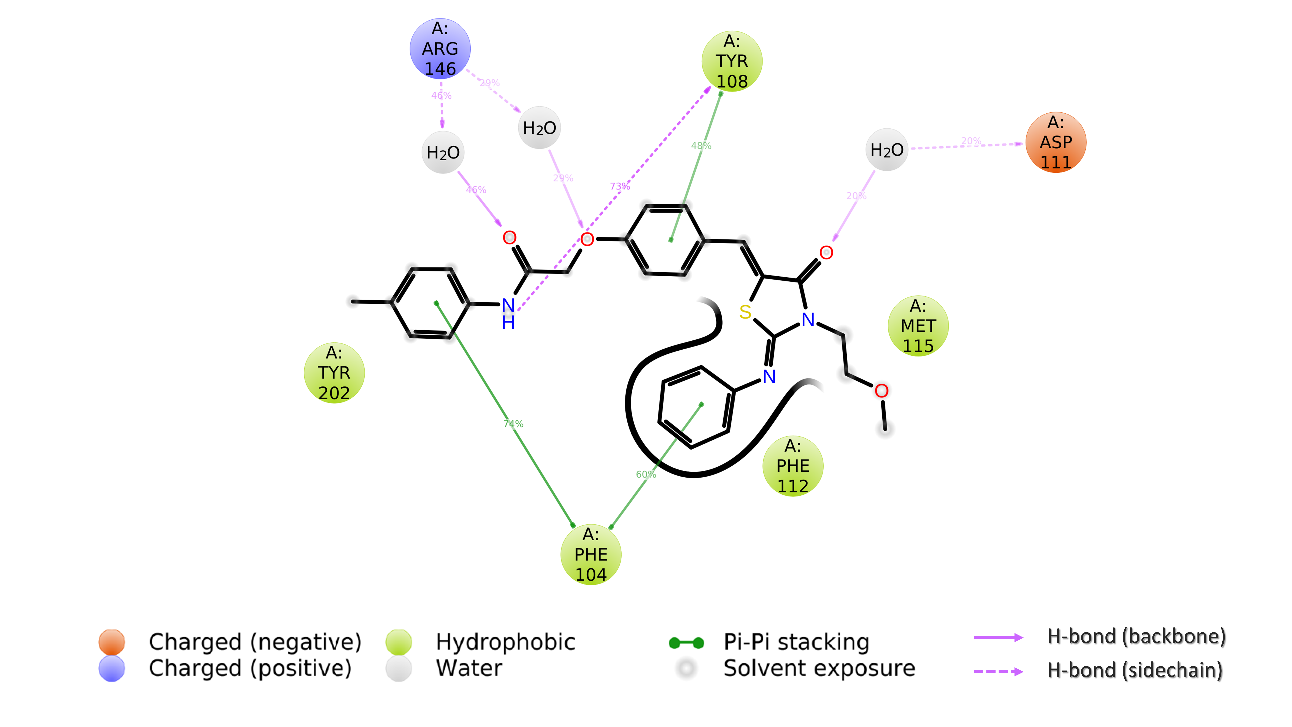

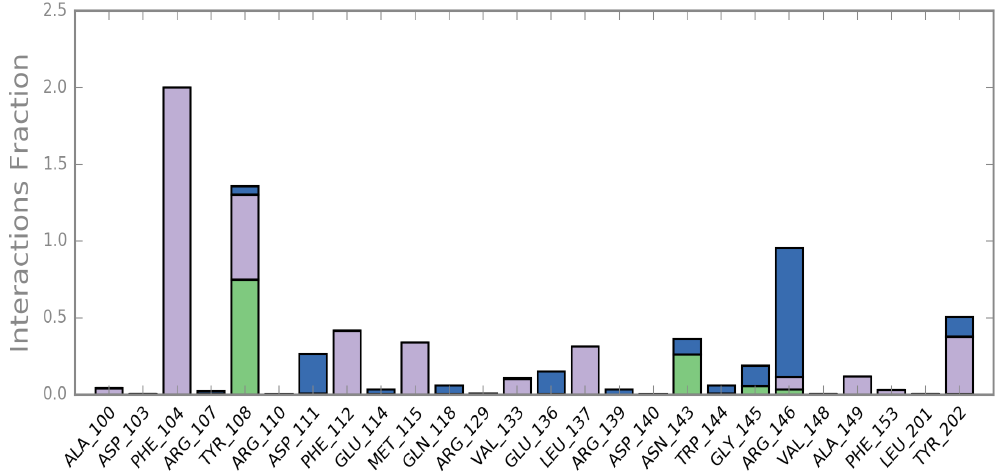

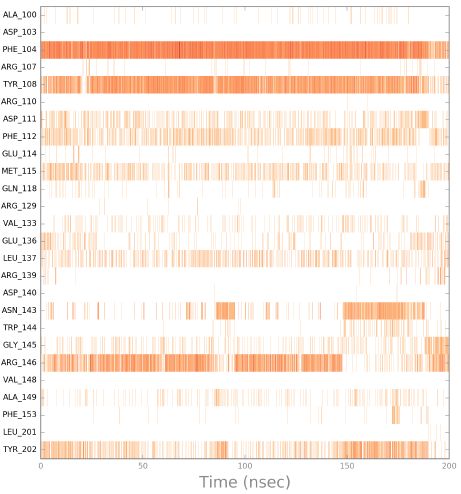


**B) C)**


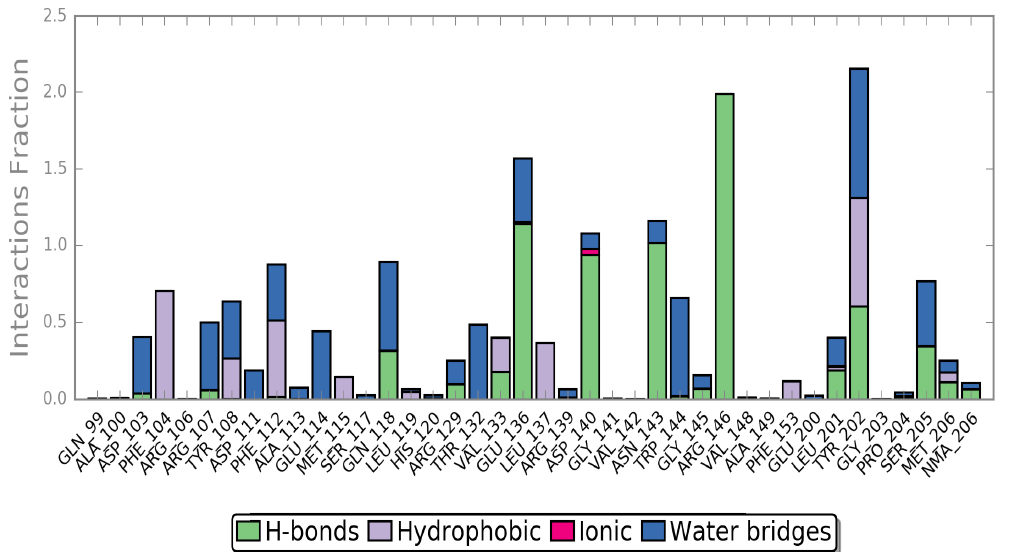


**Supplementary Figure S2** Protein-ligand interaction analysis for BAU-243 at Bcl-2 binding groove throughout MD simulation a) 2D interaction diagram with to what percentage of the simulation time the displayed interactions maintained are indicated. Cut-off value was taken as 20%. b) Histogram of non-covalent interactions, normalized over the course of trajectory. Values higher than 1.0 indicate protein residue make multiple contacts with the ligand. c) A timeline representation of the interactions for protein residues displayed in B. Darker colors denote the residue makes multiple contacts with the ligand.


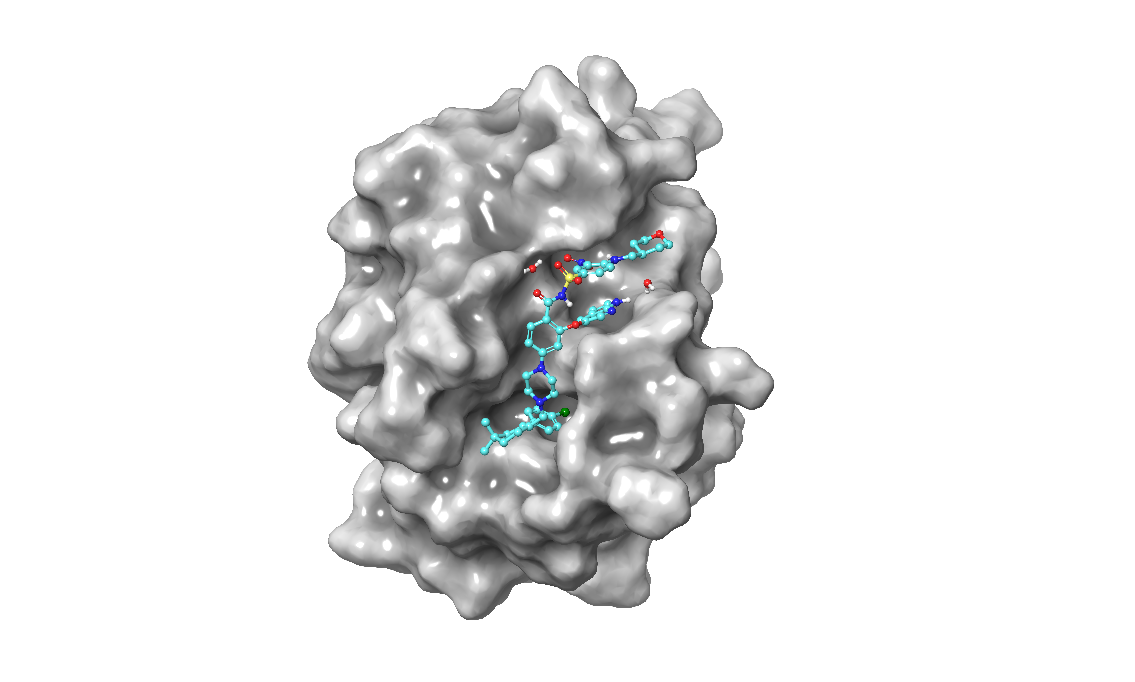


**B)**


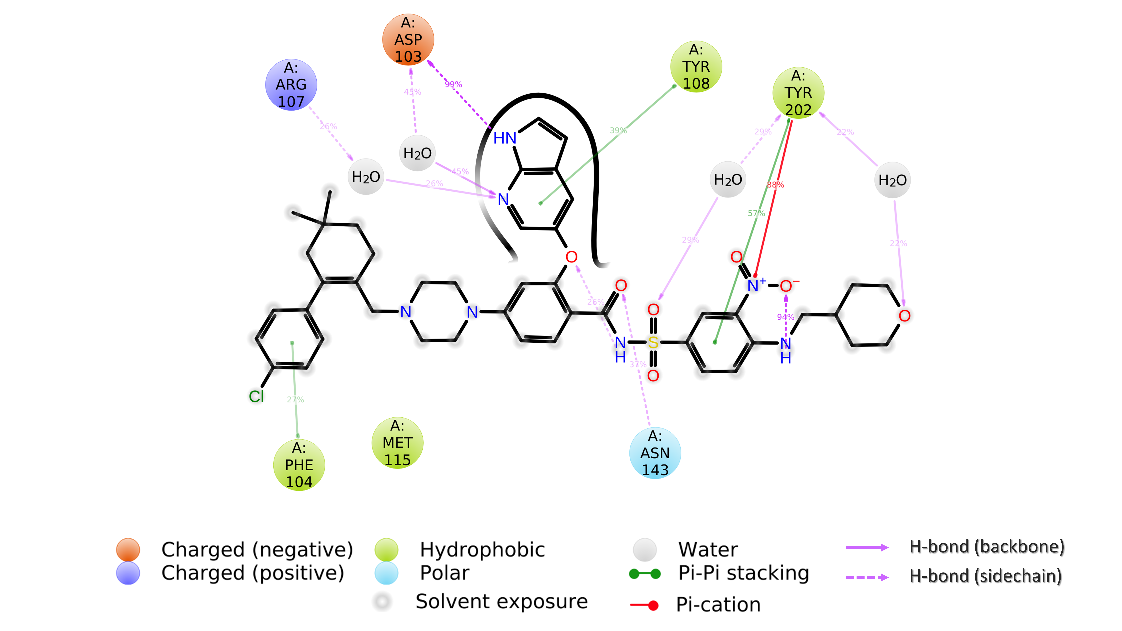


**A)**

**C) D)**


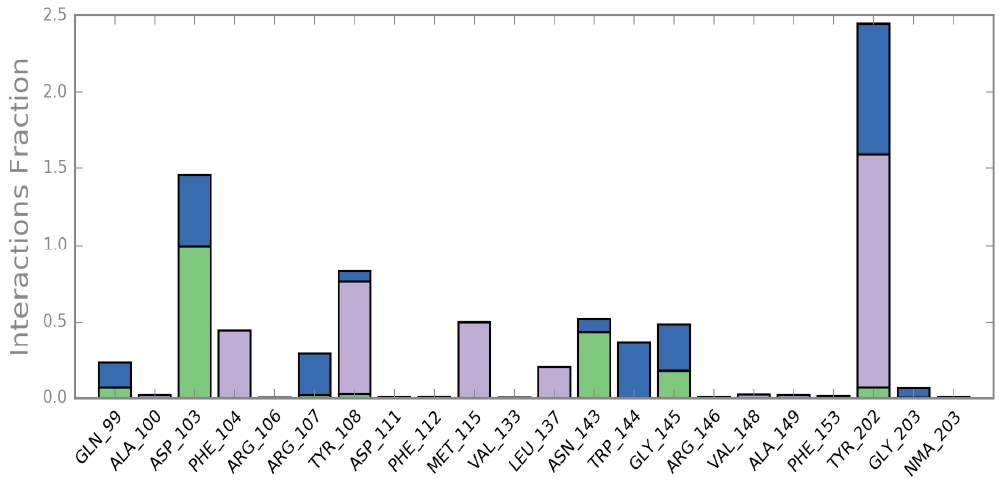

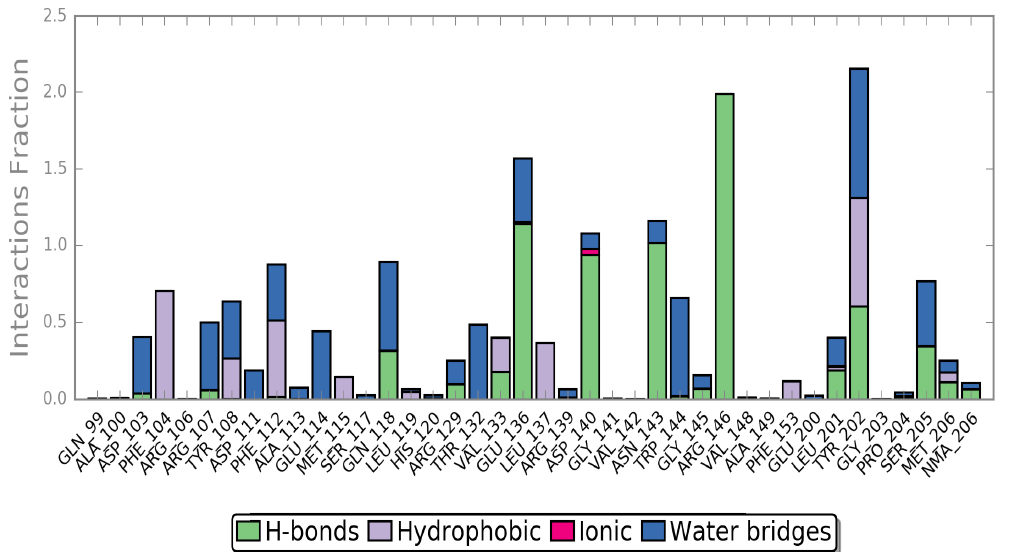

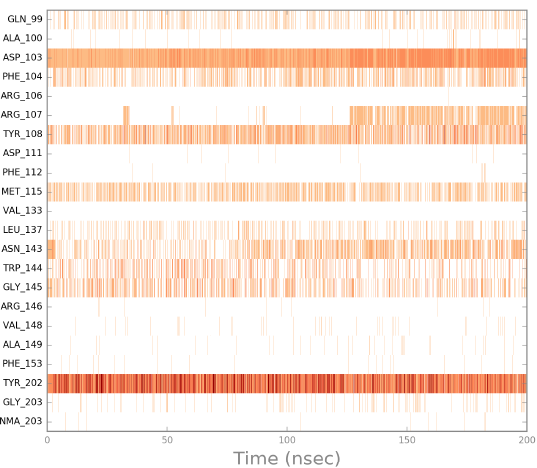


**Supplementary Figure S3** Protein-ligand interaction analysis for ABT-199 at Bcl-2 binding groove throughout MD simulation a) 2D interaction diagram with to what percentage of the simulation time the displayed interactions maintained are indicated. Cut-off value was taken as 20%. b) 3D surface display of Bcl-2. Water molecules and ABT-199 displayed with ball and stick representation with C atoms colored in dark cyan, O atoms in red, N atoms dark blue, and polar H atoms in white. c) Histogram of non-covalent interactions, normalized over the course of trajectory. Values higher than 1.0 indicate protein residue make multiple contacts with the ligand. d) A timeline representation of the interactions for protein residues displayed in B. Darker colors denote the residue makes multiple contacts with the ligand.


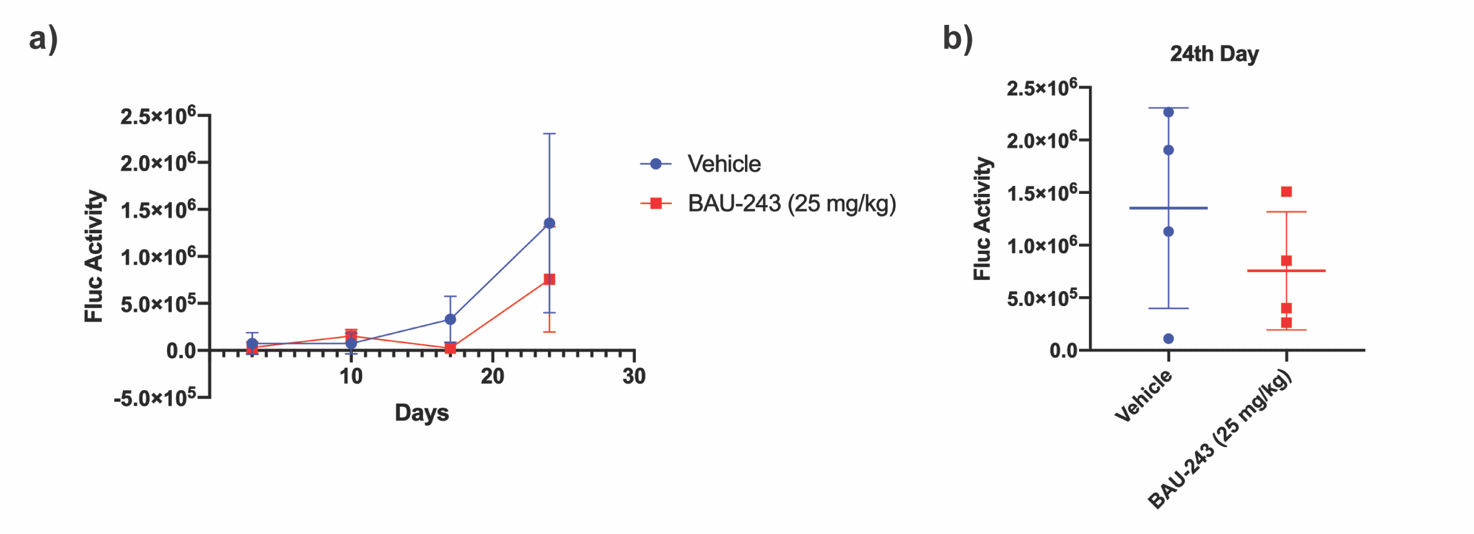


**Supplementary Figure S4** Firefly luciferase activity of BAU-243 treated animals visualized for 24 days. a) Tumor growth Fluc activity of Vehicle and BAU-243 (25mg/kg) treated mice. b) Fluc activity of each individual animal in each group (n=4) on 24th day.

**Supplementary Table S1** qPCR Primers List

| Gene Name | Forward Primer | Reverse Primer |
| --- | --- | --- |
| *BCL2* | TCGCCCTGTGGATGACTGA | CAGAGACAGCCAGGAGAAATCA |
| *BECN1* | CTGGACACTCAGCTCAACGTCA | CTCTAGTGCCAGCTCCTTTAGC |
| *MAP1LC3B* | GAGAAGCAGCTTCCTGTTCTGG | GTGTCCGTTCACCAACAGGAAG |
| *ATG5* | GCAGATGGACAGTTGCACACAC | GAGGTGTTTCCAACATTGGCTCA |

**Supplementary Table S2** Western Blotting Antibody List

| Target Name | Vendor / Catalog Number | | | Dilution | |
| --- | --- | --- | --- | --- | --- |
| PARP | | Cell Signaling Technology / #9542 | 1:500 | |  |
| p53 | | BD Biosciences / #554166 | 1:500 | |  |
| Caspase 9 | | Cell Signaling Technology / #9502 | 1:500 | |  |
| Caspase 3 | | Cell Signaling Technology / #9662S | 1:500 | |  |
| Lc3b | | Novus Biologicals / #NB100-2220SS | 1:1000 | |  |
| GAPDH | | Santa Cruz Biotechnology / #sc-32233 | 1:1000 | |  |
| Anti-rabbit IgG | | Cell Signaling Technology / #7074S | 1:7000 | |  |
| Anti-mouse IgG | | Cell Signaling Technology / #7076S | 1:7000 | |  |

**Supplementary Table S3** Average MM/GBSA scores with standard deviations calculated from trajectory files

| **Name** | **MMGBSA (kcal/mol)** | **# heavy atoms** | **Ligand Efficiency (kcal/mol)** |
| --- | --- | --- | --- |
| BH3 of Beclin 1 | -191.15 ± 11.33 | 162 | -1.18 ± 0.07 |
| BAU-243 | -100.16 ± 7.50 | 36 | -2.78 ± 0.21 |
| ABT-199 | -119.33 ± 4.66 | 61 | -1.96 ± 0.08 |
