# Supplementary material for: A novel BH3 mimetic Bcl-2 inhibitor promotes autophagic cell death and reduces in vivo Glioblastoma tumor growth
Source: Cell Death Discov. 2022 Oct 29;8:433. doi: 10.1038/s41420-022-01225-9 (PMC9617882; doi:10.1038/s41420-022-01225-9)
Supplement: Supplementary file 1 — Authors consent to addition of Dr. Asuman Celebi as co-author to this manuscript [file 41420_2022_1225_MOESM1_ESM.pdf]

# AUTHOR CONSENTS

**Kimden:** **Şeyma Çalış** seymaozkanca@gmail.com
**Konu:** Re: CDDISCOVERY-22-4841R2 Initial Quality Check
**Tarih:** 12 Ekim 2022 20:02
**Kime:** Timucin AVSAR timucin.avsar@med.bau.edu.tr
**Bilgi:** Eda Tahir Turanli edatahir@gmail.com, Turker KILIC turker.kilic@med.bau.edu.tr, Serdar Durdagi, Ph.D. serdar.durdagi@med.bau.edu.tr, Ozlem YAPICIER ozlem.yapicier@med.bau.edu.tr, Berna DOGAN berna.dogan@med.bau.edu.tr

---

Dear Dr. Avsar,

I confirm that Dr. Asuman Celebi contributed to this study and I agree to addition of her name in manuscript.

Sincerely,

Seyma CALIS, PhD

> On Oct 12, 2022, at 12:22 PM, Timucin AVSAR <timucin.avsar@med.bau.edu.tr> wrote:
>
> Dear Authors;
>
> Regarding the following mail below, i would like to get your consent about addition of Dr. Asuman Celebi as an author to our manuscript. Dr. Asuman contributed to our manuscript during revision studies.
>
> If you agree on addition of Dr. Asuman Celebi as an author to manuscript, please confirm it by replying this email.
>
> Best Regards..
>
> Timuçin Avşar (PhD)
> Assistant Professor
> Bahçeşehir University, School of Medicine
> Department of Medical Biology
>
> timucin.avsar@med.bau.edu.tr
> Office: 0090 216 579 8245
> Mobile: 0090 533 731 4937
>
> <50yil-signature.png><bau-signature.png>
>
>> İleti başlangıcı:
>>
>> **Kimden:** cddiscovery@springernature.com
>> **Konu: CDDISCOVERY-22-4841R2 Initial Quality Check**
>> **Tarih:** 12 Ekim 2022 18:13:21 GMT+3
>> **Kime:** timucin.avsar@med.bau.edu.tr
>> **Yanıt Adresi:** cddiscovery@springernature.com
>>
>> Manuscript Number: CDDISCOVERY-22-4841R2
>>
>> Title: A novel BH3 mimetic Bcl-2 inhibitor promotes autophagic cell death and reduces in vivo Glioblastoma tumor growth
>>
>> Authors: Timucin Avsar, Seyma Calis, Berna Dogan, Serdar Durdagi, Ozlem Yapicier, Turker Kilic, Eda Tahir Turanli, and Asuman Celebi
>>
>> Dear Dr Avsar,
>>
>> After carrying out a quality check of your manuscript submitted to *Cell Death Discovery*, it has come to our attention that the following points need to be addressed before we are able to pass it onto the Editors for their consideration:
>>
>> 1. Please make sure you include a data availability statement in your Article file under its own subheading.
>>
>> 2. It has come to our attention that your most recent author list differs from the one in your original submission. Please request agreement from all authors including additions and deletions, these can be collected in the following way:
>>
>> Email your co-authors with the change, and ask them to reply to your email confirming that they agree to these changes. Once you have collected these replies, please combine all of the co-authors' email responses in one document and upload this file to your submission.
>>
>> Your paper is now available for you to edit, you may access via the following link:
>>
>> https://mts-cddiscovery.nature.com/cgi-bin/main.plex?el=A6Tf1CEk3D5NmM3F3A9ftdIlxRub7aGWCCX3nayC4zxgZ

(Press/Click on the above link to be automatically sent to your journal home page.)

Please make the correction(s) as specified above and resubmit your paper following the same steps as before. Please refer to our Author Instructions (journal-specific link) for further information. If you have any questions, please do not hesitate to contact the Editorial Office at cddiscovery@springernature.com.

Yours sincerely,

Editorial Office
Cell Death & Discovery
cddiscovery@springernature.com

This email has been sent through the Springer Nature Manuscript Tracking System NY-610A-NPG&MTS

*Confidentiality Statement:*

*This e-mail is confidential and subject to copyright. Any unauthorised use or disclosure of its contents is prohibited. If you have received this email in error please notify our Manuscript Tracking System Helpdesk team at http://platformsupport.nature.com .*

*Details of the confidentiality and pre-publicity policy may be found here http://www.nature.com/authors/policies/confidentiality.html*

Privacy Policy | Update Profile

**Kimden:** **Berna DOGAN** berna.dogan@med.bau.edu.tr
**Konu:** Re: CDDISCOVERY-22-4841R2 Initial Quality Check
**Tarih:** 13 Ekim 2022 15:08
**Kime:** seymaozkanca seymaozkanca@gmail.com
**Bilgi:** Timucin AVSAR timucin.avsar@med.bau.edu.tr, Eda Tahir Turanli edatahir@gmail.com, Turker KILIC turker.kilic@med.bau.edu.tr, Serdar Durdagi, Ph.D. serdar.durdagi@med.bau.edu.tr, Ozlem YAPICIER ozlem.yapicier@med.bau.edu.tr

BD

---

Dear Dr. Avşar,

I confirm that Dr. Asuman Celebi contributed to this study and I agree with the addition of her name in manuscript.

Best regards,

Dr. Berna Dogan,
Bahcesehir University

> Şeyma Çalış <seymaozkanca@gmail.com> şunları yazdı (12 Eki 2022 20:02):
>
> Dear Dr. Avsar,
>
> I confirm that Dr. Asuman Celebi contributed to this study and I agree to addition of her name in manuscript.
>
> Sincerely,
>
> Seyma CALIS, PhD
>
>> On Oct 12, 2022, at 12:22 PM, Timucin AVSAR <timucin.avsar@med.bau.edu.tr> wrote:
>>
>> Dear Authors;
>>
>> Regarding the following mail below, i would like to get your consent about addition of Dr. Asuman Celebi as an author to our manuscript. Dr. Asuman contributed to our manuscript during revision studies.
>>
>> If you agree on addition of Dr. Asuman Celebi as an author to manuscript, please confirm it by replying this email.
>>
>> Best Regards..
>>
>> Timuçin Avşar (PhD)
>> Assistant Professor
>> Bahçeşehir University, School of Medicine
>> Department of Medical Biology
>>
>> timucin.avsar@med.bau.edu.tr
>> Office: 0090 216 579 8245
>> Mobile: 0090 533 731 4937
>>
>> <50yil-signature.png><bau-signature.png>
>>
>>> İleti başlangıcı:
>>>
>>> **Kimden:** cddiscovery@springernature.com
>>> **Konu: CDDISCOVERY-22-4841R2 Initial Quality Check**
>>> **Tarih:** 12 Ekim 2022 18:13:21 GMT+3
>>> **Kime:** timucin.avsar@med.bau.edu.tr
>>> **Yanıt Adresi:** cddiscovery@springernature.com
>>>
>>> Manuscript Number: CDDISCOVERY-22-4841R2
>>>
>>> Title: A novel BH3 mimetic Bcl-2 inhibitor promotes autophagic cell death and reduces in vivo Glioblastoma tumor growth
>>>
>>> Authors: Timucin Avsar, Seyma Calis, Berna Dogan, Serdar Durdagi, Ozlem Yapicier, Turker Kilic, Eda Tahir Turanli, and Asuman Celebi
>>>
>>> Dear Dr Avsar,
>>>
>>> After carrying out a quality check of your manuscript submitted to *Cell Death Discovery*, it has come to our attention that [illegible]

the following points need to be addressed before we are able to pass it onto the Editors for their consideration:

1. Please make sure you include a data availability statement in your Article file under its own subheading.

2. It has come to our attention that your most recent author list differs from the one in your original submission. Please request agreement from all authors including additions and deletions, these can be collected in the following way:

Email your co-authors with the change, and ask them to reply to your email confirming that they agree to these changes. Once you have collected these replies, please combine all of the co-authors' email responses in one document and upload this file to your submission.

Your paper is now available for you to edit, you may access via the following link:

https://mts-cddiscovery.nature.com/cgi-bin/main.plex?el=A6Tf1CEk3D5NmM3F3A9ftdlIxRub7aGWCCX3nayC4zxgZ

(Press/Click on the above link to be automatically sent to your journal home page.)

Please make the correction(s) as specified above and resubmit your paper following the same steps as before. Please refer to our Author Instructions (journal-specific link) for further information. If you have any questions, please do not hesitate to contact the Editorial Office at cddiscovery@springernature.com.

Yours sincerely,

Editorial Office
Cell Death & Discovery
cddiscovery@springernature.com

This email has been sent through the Springer Nature Manuscript Tracking System NY-610A-NPG&MTS

*Confidentiality Statement:*

*This e-mail is confidential and subject to copyright. Any unauthorised use or disclosure of its contents is prohibited. If you have received this email in error please notify our Manuscript Tracking System Helpdesk team at http://platformsupport.nature.com .*

*Details of the confidentiality and pre-publicity policy may be found here http://www.nature.com/authors/policies/confidentiality.html*

Privacy Policy | Update Profile

**Kimden:** **Serdar Durdagi, Ph.D.** serdar.durdagi@med.bau.edu.tr
**Konu:** Re: CDDISCOVERY-22-4841R2 Initial Quality Check
**Tarih:** 13 Ekim 2022 15:32
**Kime:** Berna DOGAN berna.dogan@med.bau.edu.tr, seymaozkanca seymaozkanca@gmail.com
**Bilgi:** Timucin AVSAR timucin.avsar@med.bau.edu.tr, Eda Tahir Turanli edatahir@gmail.com, Turker KILIC turker.kilic@med.bau.edu.tr, Ozlem YAPICIER ozlem.yapicier@med.bau.edu.tr

SD

---

Dear Dr. Avşar,

I confirm that Dr. Asuman Celebi contributed to this study and I agree with the addition of her name in manuscript.

Best regards,

Prof Dr Serdar DURDAGI
Bahcesehir University


-----------------------------------------------------------------------------
Serdar DURDAĞI, Ph.D.
Professor
Dean of School of Pharmacy, BAU

Head of Department of Basic Medical Sciences, School of Medicine, BAU.
Head of Department of Biophysics, School of Medicine, BAU.

"All Life" journal
Computational Life Sciences, Bioinformatics and System Biology
Section Editor

Durdagi Research Group (DRG)
www.durdagilab.com

Bahcesehir University
School of Medicine
Department of Biophysics
Istanbul, Turkey
Phone: +90-216-579-8217
E-mail: serdar.durdagi@med.bau.edu.tr

Prof. Dr. Serdar DURDAĞI
Bahçeşehir Üniversitesi Eczacılık Fakültesi Dekanı

Bahçeşehir Üniversitesi Tıp Fakültesi, Temel Tıp Bilimleri Bölüm Başkanı
Bahçeşehir Üniversitesi Tıp Fakültesi, Biyofizik Anabilim Dalı Başkanı

Durdagi Research Group (DRG)
www.durdagilab.com

Bahçeşehir Üniversitesi, Tıp Fakültesi
Temel Tıp Bilimleri Bölümü, Biyofizik A.B.D.
Sahrayı Cedid Mah. Batman Sok. No:66-68
Yenisahra, Kadıköy, İstanbul
Tel: 0216-579-8217
-----------------------------------------------------------------------------


________________________________________
From: Berna DOGAN <berna.dogan@med.bau.edu.tr>
Sent: Thursday, October 13, 2022 3:08 PM
To: seymaozkanca
Cc: Timucin AVSAR; Eda Tahir Turanli; Turker KILIC; Serdar Durdagi, Ph.D.; Ozlem YAPICIER
Subject: Re: CDDISCOVERY-22-4841R2 Initial Quality Check

Dear Dr. Avşar,

I confirm that Dr. Asuman Celebi contributed to this study and I agree with the addition of her name in manuscript.

Best regards,

Dr. Berna Dogan,
Bahcesehir University

Şeyma Çalış <seymaozkanca@gmail.com> şunları yazdı (12 Eki 2022 20:02):

Dear Dr. Avsar,

I confirm that Dr. Asuman Celebi contributed to this study and I agree to addition of her name in manuscript.

Sincerely,

Seyma CALIS, PhD

On Oct 12, 2022, at 12:22 PM, Timucin AVSAR <timucin.avsar@med.bau.edu.tr<mailto:timucin.avsar@med.bau.edu.tr>> wrote:

Dear Authors;
Regarding the following mail below, i would like to get your consent about addition of Dr. Asuman Celebi as an author to our manuscript. Dr. Asuman contributed to our manuscript during revision studies.
If you agree on addition of Dr. Asuman Celebi as an author to manuscript, please confirm it by replying this email.
Best Regards..

Timuçin Avşar (PhD)
Assistant Professor
Bahçeşehir University, School of Medicine
Department of Medical Biology

timucin.avsar@med.bau.edu.tr<mailto:timucin.avsar@med.bau.edu.tr>
Office: 0090 216 579 8245
Mobile: 0090 533 731 4937

<50yil-signature.png><bau-signature.png>

İleti başlangıcı:

Kimden: cddiscovery@springernature.com<mailto:cddiscovery@springernature.com>
Konu: CDDISCOVERY-22-4841R2 Initial Quality Check
Tarih: 12 Ekim 2022 18:13:21 GMT+3
Kime: timucin.avsar@med.bau.edu.tr<mailto:timucin.avsar@med.bau.edu.tr>
Yanıt Adresi: cddiscovery@springernature.com<mailto:cddiscovery@springernature.com>

Manuscript Number: CDDISCOVERY-22-4841R2

Title: A novel BH3 mimetic Bcl-2 inhibitor promotes autophagic cell death and reduces in vivo Glioblastoma tumor growth

Authors: Timucin Avsar, Seyma Calis, Berna Dogan, Serdar Durdagi, Ozlem Yapicier, Turker Kilic, Eda Tahir Turanli, and Asuman Celebi

Dear Dr Avsar,

After carrying out a quality check of your manuscript submitted to Cell Death Discovery, it has come to our attention that the following points need to be addressed before we are able to pass it onto the Editors for their consideration:

1. Please make sure you include a data availability statement in your Article file under its own subheading.

2. It has come to our attention that your most recent author list differs from the one in your original submission. Please request agreement from all authors including additions and deletions, these can be collected in the following way:

Email your co-authors with the change, and ask them to reply to your email confirming that they agree to these changes. Once you have collected these replies, please combine all of the co-authors' email responses in one document and upload this file to your submission.

Your paper is now available for you to edit, you may access via the following link:

<https://mts-cddiscovery.nature.com/cgi-bin/main.plex?el=A6Tf1CEk3D5NmM3F3A9ftdIlxRub7aGWCCX3nayC4zxgZ>https://mts-cddiscovery.nature.com/cgi-bin/main.plex?el=A6Tf1CEk3D5NmM3F3A9ftdIlxRub7aGWCCX3nayC4zxgZ

(Press/Click on the above link to be automatically sent to your journal home page.)

Please make the correction(s) as specified above and resubmit your paper following the same steps as before. Please refer to our Author Instructions (journal-specific link) for further information. If you have any questions, please do not hesitate to contact

the Editorial Office at cddiscovery@springernature.com<mailto:cddiscovery@springernature.com>.

Yours sincerely,

Editorial Office
Cell Death & Discovery
cddiscovery@springernature.com<mailto:cddiscovery@springernature.com>

This email has been sent through the Springer Nature Manuscript Tracking System NY-610A-NPG&MTS

Confidentiality Statement:

This e-mail is confidential and subject to copyright. Any unauthorised use or disclosure of its contents is prohibited. If you have received this email in error please notify our Manuscript Tracking System Helpdesk team at <http://platformsupport.nature.com/> http://platformsupport.nature.com<http://platformsupport.nature.com/> .

Details of the confidentiality and pre-publicity policy may be found here <http://www.nature.com/authors/policies/confidentiality.html> http://www.nature.com/authors/policies/confidentiality.html

Privacy Policy<http://www.nature.com/info/privacy.html> | Update Profile<https://mts-cdd.nature.com/>

**Kimden:** Ozlem YAPICIER ozlem.yapicier@med.bau.edu.tr
**Konu:** RE: CDDISCOVERY-22-4841R2 Initial Quality Check
**Tarih:** 14 Ekim 2022 08:49
**Kime:** Timucin AVSAR timucin.avsar@med.bau.edu.tr

OY

Dear Timuçin Avşar,
I confirm that Dr. Asuman Celebi contributed to this study and I agree to addition of her name in manuscript.
Özlem Yapıcıer, M.D.

---

**From:** Timucin AVSAR <timucin.avsar@med.bau.edu.tr>
**Sent:** Wednesday, October 12, 2022 7:22 PM
**To:** Eda Tahir Turanli <edatahir@gmail.com>; Turker KILIC <turker.kilic@med.bau.edu.tr>; Serdar Durdagi, Ph.D. <serdar.durdagi@med.bau.edu.tr>; Ozlem YAPICIER <ozlem.yapicier@med.bau.edu.tr>; seymaozkanca <seymaozkanca@gmail.com>; Berna DOGAN <berna.dogan@med.bau.edu.tr>
**Subject:** Fwd: CDDISCOVERY-22-4841R2 Initial Quality Check

Dear Authors;
Regarding the following mail below, i would like to get your consent about addition of Dr. Asuman Celebi as an author to our manuscript. Dr. Asuman contributed to our manuscript during revision studies.
If you agree on addition of Dr. Asuman Celebi as an author to manuscript, please confirm it by replying this email.
Best Regards..

Timuçin Avşar (PhD)
Assistant Professor
Bahçeşehir University, School of Medicine
Department of Medical Biology

timucin.avsar@med.bau.edu.tr
Office: 0090 216 579 8245
Mobile: 0090 533 731 4937

İleti başlangıcı:

**Kimden:** cddiscovery@springernature.com
**Konu: CDDISCOVERY-22-4841R2 Initial Quality Check**
**Tarih:** 12 Ekim 2022 18:13:21 GMT+3
**Kime:** timucin.avsar@med.bau.edu.tr
**Yanıt Adresi:** cddiscovery@springernature.com

Manuscript Number: CDDISCOVERY-22-4841R2

Title: A novel BH3 mimetic Bcl-2 inhibitor promotes autophagic cell death and reduces in vivo Glioblastoma tumor growth

Authors: Timucin Avsar, Seyma Calis, Berna Dogan, Serdar Durdagi, Ozlem Yapicier, Turker Kilic, Eda Tahir Turanli, and Asuman Celebi

Dear Dr Avsar,

After carrying out a quality check of your manuscript submitted to *Cell Death Discovery*, it has come to our attention that the following points need to be addressed before we are able to pass it onto the Editors for their consideration:

1. Please make sure you include a data availability statement in your Article file under its own subheading.

2. It has come to our attention that your most recent author list differs from the one in your original submission. Please request agreement from all authors including additions and deletions, these can be collected in the following way:

Email your co-authors with the change, and ask them to reply to your email confirming that they agree to these changes. Once you have collected these replies, please combine all of the co-authors' email responses in one document and upload this file to your submission.

Your paper is now available for you to edit, you may access via the following link:

https://mts-cddiscovery.nature.com/cgi-bin/main.plex?el=A6Tf1CEk3D5NmM3F3A9ftdlIxRub7aGWCCX3nayC4zxgZ

(Press/Click on the above link to be automatically sent to your journal home page.)

Please make the correction(s) as specified above and resubmit your paper following the same steps as before. Please refer to our Author Instructions (journal-specific link) for further information. If you have any questions, please do not hesitate to contact the Editorial Office at cddiscovery@springernature.com.

Yours sincerely,

Editorial Office
Cell Death & Discovery
cddiscovery@springernature.com

This email has been sent through the Springer Nature Manuscript Tracking System NY-610A-NPG&MTS

*Confidentiality Statement:*

*This e-mail is confidential and subject to copyright. Any unauthorised use or disclosure of its contents is prohibited. If you have received this email in error please notify our Manuscript Tracking System Helpdesk team at http://platformsupport.nature.com .*
*Details of the confidentiality and pre-publicity policy may be found here http://www.nature.com/authors/policies/confidentiality.html*
Privacy Policy | Update Profile

**Kimden:** **Turker KILIC** turker.kilic@med.bau.edu.tr
**Konu:** Re: CDDISCOVERY-22-4841R2 Initial Quality Check
**Tarih:** 14 Ekim 2022 11:18
**Kime:** Timucin AVSAR timucin.avsar@med.bau.edu.tr

TK

**"I confirm that Dr. Asuman Celebi contributed to this study and I agree to addition of her name in manuscript.**


Türker Kılıç MD, PhD
Bahcesehir University (BAU)
School of Medicine, Dean
Professor of Neurosurgery, Chairman *Istanbul, Turkey*
*www.beyincerrahisi.net*
*+90 538 303 8585*
*turker.kilic@med.bahcesehir.edu.tr*


Eda Tahir Turanli <edatahir@gmail.com> şunları yazdı (13 Eki 2022 15:39):

Dear Dr. Avşar,

I confirm that Dr. Asuman Celebi contributed to this study and I agree with the addition of her name in the manuscript.
Kind Regards,
Eda Tahir Turanli


Eda Tahir Turanli, PhD

Professor and Chair, Department of Molecular Biology and Genetics

Faculty of Arts and Sciences, Acibadem University

Kerem Aydinlar Campus, Kayisdagi Cad. No 32, 34752

Istanbul, Turkey

https://www.acibadem.edu.tr

Social Media: mbg_acu

Tel: +90 216 500 4270; GSM: +90 532 524 6521


On Wed, Oct 12, 2022 at 7:20 PM Timucin AVSAR <timucin.avsar@med.bau.edu.tr> wrote:

Sayın hocalarım merhaba,

Cell Death Discovery dergisine gönderdiğimiz yayın kabul edildi.

Revizyonlar sırasında deneyleri gerçekleştiren Dr. Asuman Çelebi'nin makale yazarları listesine sonradan eklenmesi nedeniyle, dergi tüm yazarlardan mail olarak bir uygunluk yazısı almamızı istiyor.

Sizlerden ricam, bir sonraki atacağım maile aşağıdaki ifadeyi kullanarak ya da istediğiniz şekilde bir ifade kullanarak yanıt vermeniz. Verdiğiniz yanıt maillerini bir doküman yaparak sisteme yükleyeceğim.

Destekleriniz için tekrar teşekkürler.

Saygılarımla..

**"I confirm that Dr. Asuman Celebi contributed to this study and I agree to addition of her name in manuscript."**


Dr. Öğr. Üyesi Timuçin Avşar
Bahçeşehir Üniversitesi Tıp Fakültesi, Tıbbi Biyoloji ABD

timucin.avsar@med.bau.edu.tr
Ofis: 0 216 579 8245

Ofis: 0 216 579 8245
Cep: 0 533 731 4937

İleti başlangıcı:

**Kimden:** cddiscovery@springernature.com
**Konu:** **CDDISCOVERY-22-4841R2 Initial Quality Check**
**Tarih:** 12 Ekim 2022 18:13:21 GMT+3
**Kime:** timucin.avsar@med.bau.edu.tr
**Yanıt Adresi:** cddiscovery@springernature.com

Manuscript Number: CDDISCOVERY-22-4841R2

Title: A novel BH3 mimetic Bcl-2 inhibitor promotes autophagic cell death and reduces in vivo Glioblastoma tumor growth

Authors: Timucin Avsar, Seyma Calis, Berna Dogan, Serdar Durdagi, Ozlem Yapicier, Turker Kilic, Eda Tahir Turanli, and Asuman Celebi

Dear Dr Avsar,

After carrying out a quality check of your manuscript submitted to *Cell Death Discovery*, it has come to our attention that the following points need to be addressed before we are able to pass it onto the Editors for their consideration:

1. Please make sure you include a data availability statement in your Article file under its own subheading.

2. It has come to our attention that your most recent author list differs from the one in your original submission. Please request agreement from all authors including additions and deletions, these can be collected in the following way:

Email your co-authors with the change, and ask them to reply to your email confirming that they agree to these changes. Once you have collected these replies, please combine all of the co-authors' email responses in one document and upload this file to your submission.

Your paper is now available for you to edit, you may access via the following link:

https://mts-cddiscovery.nature.com/cgi-bin/main.plex?el=A6Tf1CEk3D5NmM3F3A9ftdIlxRub7aGWCCX3nayC4zxgZ

(Press/Click on the above link to be automatically sent to your journal home page.)

Please make the correction(s) as specified above and resubmit your paper following the same steps as before. Please refer to our Author Instructions (journal-specific link) for further information. If you have any questions, please do not hesitate to contact the Editorial Office at cddiscovery@springernature.com.

Yours sincerely,

Editorial Office
Cell Death & Discovery
cddiscovery@springernature.com

This email has been sent through the Springer Nature Manuscript Tracking System NY-610A-NPG&MTS

*Confidentiality Statement:*

*This e-mail is confidential and subject to copyright. Any unauthorised use or disclosure of its contents is prohibited. If you have received this email in error please notify our Manuscript Tracking System Helpdesk team at http://platformsupport.nature.com .*

*Details of the confidentiality and pre-publicity policy may be found here http://www.nature.com/authors/policies/confidentiality.html*

Privacy Policy | Update Profile

**Kimden:** Eda Tahir Turanli edatahir@gmail.com
**Konu:** Re: CDDISCOVERY-22-4841R2 Initial Quality Check
**Tarih:** 13 Ekim 2022 15:39
**Kime:** Timucin AVSAR timucin.avsar@med.bau.edu.tr
**Bilgi:** Turker KILIC turker.kilic@med.bau.edu.tr, Serdar Durdagi, Ph.D. serdar.durdagi@med.bau.edu.tr, Ozlem YAPICIER ozlem.yapicier@med.bau.edu.tr, seymaozkanca seymaozkanca@gmail.com, Berna DOGAN berna.dogan@med.bau.edu.tr, asumancelebi asumancelebi@yahoo.com.tr

ET

---

Dear Dr. Avşar,

I confirm that Dr. Asuman Celebi contributed to this study and I agree with the addition of her name in the manuscript.
Kind Regards,
Eda Tahir Turanli

Eda Tahir Turanli, PhD

Professor and Chair, Department of Molecular Biology and Genetics

Faculty of Arts and Sciences, Acibadem University

Kerem Aydinlar Campus, Kayisdagi Cad. No 32, 34752

Istanbul, Turkey

https://www.acibadem.edu.tr

Social Media: mbg_acu

Tel: +90 216 500 4270; GSM: +90 532 524 6521

On Wed, Oct 12, 2022 at 7:20 PM Timucin AVSAR <timucin.avsar@med.bau.edu.tr> wrote:

> Sayın hocalarım merhaba,
>
> Cell Death Discovery dergisine gönderdiğimiz yayın kabul edildi.
>
> Revizyonlar sırasında deneyleri gerçekleştiren Dr. Asuman Çelebi'nin makale yazarları listesine sonradan eklenmesi nedeniyle, dergi tüm yazarlardan mail olarak bir uygunluk yazısı almamızı istiyor.
>
> Sizlerden ricam, bir sonraki atacağım maile aşağıdaki ifadeyi kullanarak ya da istediğiniz şekilde bir ifade kullanarak yanıt vermeniz. Verdiğiniz yanıt maillerini bir doküman yaparak sisteme yükleyeceğim.
>
> Destekleriniz için tekrar teşekkürler.
>
> Saygılarımla..
>
> **"I confirm that Dr. Asuman Celebi contributed to this study and I agree to addition of her name in manuscript."**
>
> Dr. Öğr. Üyesi Timuçin Avşar
> Bahçeşehir Üniversitesi Tıp Fakültesi, Tıbbi Biyoloji ABD
>
> timucin.avsar@med.bau.edu.tr
> Ofis: 0 216 579 8245
> Cep: 0 533 731 4937
>
> BAHÇEŞEHİR UĞUR EĞİTİM KURUMLARI · BAU Bahçeşehir University
>
> > İleti başlangıcı:
> >
> > **Kimden:** cddiscovery@springernature.com
> > **Konu:** **CDDISCOVERY-22-4841R2 Initial Quality Check**
> > **Tarih:** 12 Ekim 2022 18:13:21 GMT+3
> > **Kime:** timucin.avsar@med.bau.edu.tr
> > **Yanıt Adresi:** cddiscovery@springernature.com
> >
> > Manuscript Number: CDDISCOVERY-22-4841R2

Title: A novel BH3 mimetic Bcl-2 inhibitor promotes autophagic cell death and reduces in vivo Glioblastoma tumor growth

Authors: Timucin Avsar, Seyma Calis, Berna Dogan, Serdar Durdagi, Ozlem Yapicier, Turker Kilic, Eda Tahir Turanli, and Asuman Celebi

Dear Dr Avsar,

After carrying out a quality check of your manuscript submitted to *Cell Death Discovery*, it has come to our attention that the following points need to be addressed before we are able to pass it onto the Editors for their consideration:

1. Please make sure you include a data availability statement in your Article file under its own subheading.

2. It has come to our attention that your most recent author list differs from the one in your original submission. Please request agreement from all authors including additions and deletions, these can be collected in the following way:

Email your co-authors with the change, and ask them to reply to your email confirming that they agree to these changes. Once you have collected these replies, please combine all of the co-authors' email responses in one document and upload this file to your submission.

Your paper is now available for you to edit, you may access via the following link:

https://mts-cddiscovery.nature.com/cgi-bin/main.plex?el=A6Tf1CEk3D5NmM3F3A9ftdlIxRub7aGWCCX3nayC4zxgZ

(Press/Click on the above link to be automatically sent to your journal home page.)

Please make the correction(s) as specified above and resubmit your paper following the same steps as before. Please refer to our Author Instructions (journal-specific link) for further information. If you have any questions, please do not hesitate to contact the Editorial Office at cddiscovery@springernature.com.

Yours sincerely,

Editorial Office
Cell Death & Discovery
cddiscovery@springernature.com

This email has been sent through the Springer Nature Manuscript Tracking System NY-610A-NPG&MTS

*Confidentiality Statement:*

*This e-mail is confidential and subject to copyright. Any unauthorised use or disclosure of its contents is prohibited. If you have received this email in error please notify our Manuscript Tracking System Helpdesk team at http://platformsupport.nature.com .*

*Details of the confidentiality and pre-publicity policy may be found here http://www.nature.com/authors/policies/confidentiality.html*

Privacy Policy | Update Profile
